# Influenza A virus segments five and six can harbor artificial introns allowing expanded coding capacity

**DOI:** 10.1371/journal.ppat.1009951

**Published:** 2021-09-27

**Authors:** Heather M. Froggatt, Kaitlyn N. Burke, Ryan R. Chaparian, Hector A. Miranda, Xinyu Zhu, Benjamin S. Chambers, Nicholas S. Heaton

**Affiliations:** 1 Department of Molecular Genetics and Microbiology Duke University School of Medicine Durham, North Carolina, United States of America; 2 Duke Human Vaccine Institute Duke University School of Medicine Durham, North Carolina, United States of America; Freiburg University, GERMANY

## Abstract

Influenza A viruses encode their genomes across eight, negative sense RNA segments. The six largest segments produce mRNA transcripts that do not generally splice; however, the two smallest segments are actively spliced to produce the essential viral proteins NEP and M2. Thus, viral utilization of RNA splicing effectively expands the viral coding capacity without increasing the number of genomic segments. As a first step towards understanding why splicing is not more broadly utilized across genomic segments, we designed and inserted an artificial intron into the normally nonsplicing NA segment. This insertion was tolerated and, although viral mRNAs were incompletely spliced, we observed only minor effects on viral fitness. To take advantage of the unspliced viral RNAs, we encoded a reporter luciferase gene in frame with the viral ORF such that when the intron was not removed the reporter protein would be produced. This approach, which we also show can be applied to the NP encoding segment and in different viral genetic backgrounds, led to high levels of reporter protein expression with minimal effects on the kinetics of viral replication or the ability to cause disease in experimentally infected animals. These data together show that the influenza viral genome is more tolerant of splicing than previously appreciated and this knowledge can be leveraged to develop viral genetic platforms with utility for biotechnology applications.

## Author summary

Unlike most host mRNAs, some viral mRNAs encode multiple discrete, functional proteins. One method influenza A viruses use to increase the protein products from two of their eight RNA genome segments is splicing. Splicing requires host machinery to remove part of the viral mRNA, the intron, to generate a different mRNA product. Although only certain influenza viral segments naturally splice, we were interested in whether additional segments could splice to produce multiple proteins. We inserted artificial introns harboring reporter genes into otherwise nonsplicing genomic segments of an H1N1 influenza A virus and found that this modification was well tolerated by the virus. We further demonstrated that an unrelated H3N2 influenza A virus could similarly support splicing and express a reporter protein from an artificial intron. These findings have implications for our understanding of how viruses expand their coding capacity with a limited genome. Additionally, encoding reporter proteins in spliced intronic sequences also represents a new method of generating reporter viruses requiring limited manipulation of the viral RNA.

## Introduction

RNA viruses have a limited genetic space. To expand their coding capacity, many RNA viruses use alternative translation initiation sites and ribosomal frameshifting to access alternative reading frames encoding an additional protein or RNA product [1]. While most non-retroviral RNA viruses replicate in the cytoplasm, select others, such as viruses in the *Orthomyxoviridae* and *Bornaviridae* families, enter the nucleus to replicate their genomes and transcribe viral mRNAs [2]. Nuclear replication enables viral access to another tool for diversifying their encoded proteins: the host cell splicing machinery, which can allow distinct proteins to be produced from a single transcript.

Influenza A virus (IAV) uses the splicing of segments 7/M and 8/NS to generate multiple mRNA species and multiple proteins (M1/M2 and NS1/NEP, respectively) from a single viral segment [3,4]. Beyond generating multiple proteins from a single gene, viruses also take advantage of splicing to regulate viral gene expression. During IAV infections the ratio between two proteins produced from the NS segment, unspliced NS1 and spliced NEP, is skewed towards NS1 [5] to facilitate the NS1 levels necessary to suppress host immune responses [6]. In contrast, as IAV infection progresses mRNAs produced from the M segment are spliced more often, increasing the amount of spliced M2 relative to unspliced M1 over time [7]; after contributing to viral entry the M2 ion channel is thought to primarily be required late in replication during viral assembly [8]. Furthermore, splicing dysregulation in different host environments reduces viral replication efficiency, likely as a result of alterations to viral protein ratios [9–13]. These observations together demonstrate the importance of splicing in optimizing the influenza viral replication processes.

Despite the apparent tight controls of splicing, IAV segments 7 and 8 tolerate alterations to canonical splicing regulation. For example, in addition to M1/M2 splicing, there is also a third M segment-derived transcript, mRNA3, that is conserved but goes untranslated [14]. A limited number of strains also encode an additional 3’ splice site in NS that results in the NS3 transcript and protein [15]. Another group of strains encode an additional 5’ splice site in the M segment to produce the untranslated M42 transcript [16]. These findings show that additional splicing within already spliced IAV segments is tolerable and, because these mutants occur naturally, potentially advantageous. Further, lab-generated viruses containing modified NS segments where splicing is eliminated and NS1 and NEP are “split” and separated by a 2A cleavage site are well tolerated and capable of encoding reporter proteins [17,18]. In contrast, analogous recombinant viruses “splitting” the M segment M1 and M2 sequences replicate poorly [19]. Nevertheless, recombinant IAVs that “split” both M and NS segments have been rescued, demonstrating that splicing can be eliminated from the IAV genome [19]. Thus, the importance and flexibility of splicing in IAV segments 7 and 8 are well recognized; however, the potential of splicing in additional IAV segments is generally less known.

Existing literature regarding genomic segment splicing across the *Orthomyxoviridae* family fails to reveal a clear consensus on the range or limits of viral RNA splicing. For instance, the shortest genome segments are frequently spliced in each member of the family: Segments 7 and 8 in 8-segmented IAV; segment 8 in 8-segmented influenza B virus (IBV); segments 6 and 7 in 7-segmented influenza C virus; segment 6 in 6-segmented Thogoto virus; and segment 7 in 7-segmented issavirus [20]. However, splicing in long segments has been reported as well. For example, a splicing product (PB2-S1) derived from the longest segment, segment 1/PB2, was identified in pre-2009 pandemic H1N1 IAVs [21]. Furthermore, viral genomes and transcripts are optimized during viral evolution, meaning that additional splicing is only observable when it confers an advantage; the range of segments where splicing is tolerable could differ significantly from where it is beneficial. It therefore remains unclear if normally nonsplicing viral segments can tolerate splicing and what the effects on viral biology would be.

To experimentally probe the influenza viral genome for tolerance of additional splicing, we designed artificial introns with different characteristics and inserted them into the otherwise nonsplicing NA segment. Viruses containing artificial introns were viable, and the composition of the intron itself was not a major constraint on the tolerance of artificially introduced splicing. In fact, introns harboring a full-length reporter gene were well tolerated and could functionally express protein from unspliced transcripts derived from not only the neuraminidase (NA) but also the nucleoprotein (NP) encoding genomic segment. Based on these experiments (performed in a laboratory-adapted H1N1 genetic background), we developed a set of “rules” for the insertion of artificial introns into any IAV genome; we then demonstrated the utility of this approach by generating a intronic reporter H3N2 IAV. In sum, these data reveal a general tolerance of artificially introduced splicing in normally intronless IAV genomic segments, results with implications for biotechnology applications such as the generation of reporter viral strains.

## Results

### IAV segment 6 tolerates introduction of an artificial intron

**Fig 1:**
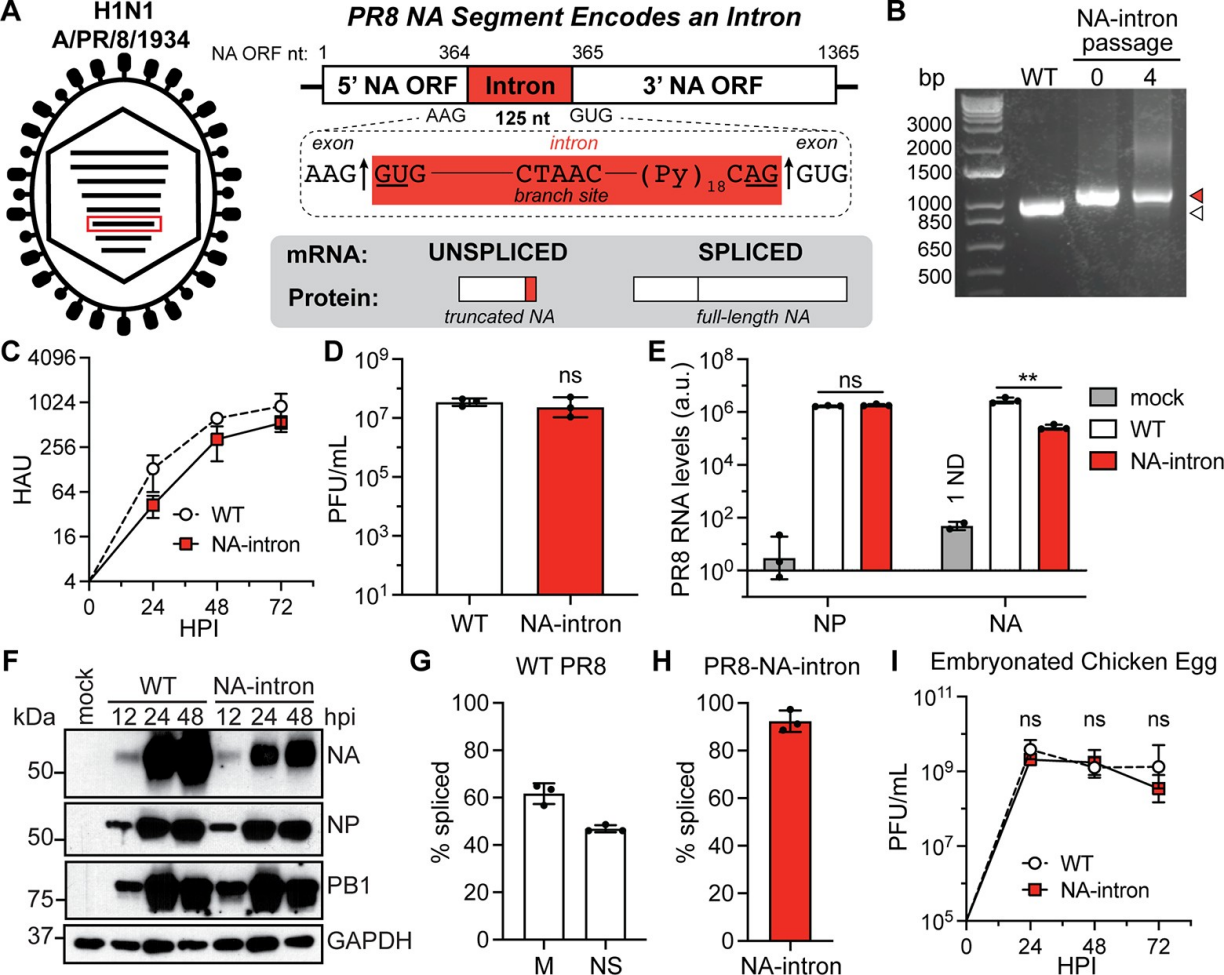
An artificial intron can be inserted into the IAV segment 6. (A) Diagram of the A/Puerto Rico/8/1934 H1N1 influenza A virus (IAV) segment 6/NA containing an intron and its protein products. ORF, open reading frame. (B) RT-PCR of WT PR8 virus (white) and PR8-NA-intron virus (red) segment 6/NA at passages 0 and 4 from serial PR8-NA-intron virus infections (MOI = 0.01, multicycle, 72h passages) on MDCK cells (representative of three independent experiments). (C) Growth kinetics of WT PR8 and PR8-NA-intron virus infections (MOI = 0.001, multicycle) on MDCK cells, measured using HA assays (mean with SD, n = 3 independent experiments). HAU, hemagglutination units; HPI, hours post-infection. (D) End point titers of WT PR8 and PR8-NA-intron virus infections (MOI = 0.01, multicycle, 72h) on MDCK cells, measured using plaque assays (mean with SD, n = 3 independent experiments, unpaired Student’s t-test). PFU, plaque forming units. (E) PR8 NA and NP RNA levels from mock, WT PR8, PR8-NA-intron virus infections (MOI = 2, single cycle, 8h) on MDCK cells, measured using one-step RT-qPCR (mean with SD, n = 3 independent experiments, unpaired Student’s t-test). Arbitrary units (a.u.) determined relative to 18S; ND, not determined. (F) Western blots for PR8 NA, NP and PB1 proteins from mock, PR8 WT, and PR8-NA-intron virus infections (MOI = 0.01, multicycle) on MDCK cells (representative of three independent experiments). GAPDH was used as a loading control. (G) PR8 segment 7/M and segment 8/NS mRNA splicing rates from WT PR8 virus infections (MOI = 2, single cycle, 8h) on MDCK cells, measured using two-step RT-qPCR (mean with SD, n = 3 independent experiments). (H) PR8 segment 6/NA-intron mRNA splicing rates from PR8-NA-intron virus infections (MOI = 2, single cycle, 8h) on MDCK cells, measured using two-step RT-qPCR (mean with SD, n = 3 independent experiments). (I) Growth kinetics of WT PR8 and PR8-NA-intron virus infections (100PFU) in embryonated chicken eggs, measured using plaque assays (mean with SD, n = 3 eggs per group, unpaired Student’s t-test). For all panels: *P < 0.05, **P < 0.001, and ns = not significant.

To investigate whether normally nonsplicing viral RNAs can tolerate splicing during a viral infection, we aimed to introduce an artificial intron via reverse genetics. To accomplish this goal we selected a constitutively spliced intron sequence [22] with the idea that, after insertion into the viral segment, the dominant mRNA species would encode a functional viral protein, rather than the intron-retained, nonfunctional version. We selected the H1N1 A/Puerto Rico/8/1934 strain segment 6 (which encodes the viral neuraminidase, NA) as the intron target because it is the next shortest segment after the spliced segments 7 and 8 and the increased genomic segment length would not exceed the length of the longest viral segments. To generate the intron-containing segment, we identified a six-nucleotide sequence, “AAGGUG,” within the NA coding region. We inserted the constitutively spliced intron sequence after the PR8 NA encoded “AAG,” forming part of a splice donor site, and before the encoded “GUG,” forming part of a splice acceptor site (**Fig 1A**). As designed, the spliced version of this PR8 NA-intron mRNA should be identical to the wild-type (WT) PR8 NA mRNA. The unspliced version of the PR8 NA-intron mRNA retains the intron and encodes a stop codon, resulting in a truncated protein product (**Fig 1A**). We rescued this virus in the PR8 background (PR8-NA-intron) and determined the stability of our PR8-NA-intron virus over four serial passages on MDCK cells and observed no loss in segment length (**Fig 1B**). We also found it grew to high titers without a significant growth defect compared to WT PR8 under multicycle growth conditions on MDCK cells (**Fig 1C and 1D**). Together, these findings demonstrate that IAV PR8 tolerates the introduction of a highly spliced intron in segment 6.

The presence or absence of introns in influenza virus mRNAs is recognized to impact their transport and translation [4]. To determine if the addition of an intron impacted the transcription, replication, or translation of NA, we measured the RNA (using an assay that would not discriminate between mRNA, vRNA, and cRNA) and protein levels from WT PR8 or PR8-NA-intron virus-infected MDCK cells. We found there was a modest reduction in NA RNA expression levels and a corresponding decrease in NA protein levels between our PR8-NA-intron virus and the WT PR8 virus (**Fig 1E and 1F**).

We designed the PR8 NA-intron segment to be highly spliced while endogenous influenza intronic sequences are often retained to reflect the protein needs of a replicating virus [5]. Therefore, we expected our introduced segment 6 intron to be spliced at a higher rate than the endogenous introns in IAV segments 7 and 8. We observed splicing rates around 60% and 40% for the WT PR8 M and NS segments, respectively, during a WT PR8 virus infection in MDCK cells (**Figs 1G and S1A–S1C**). In contrast, our constitutively spliced artificial intron was spliced in about 90% of NA-intron mRNA transcripts (**Figs 1H and S1D**). Time also dictates IAV mRNA splicing rates and protein balance during infection; early in infection, M1 is expressed more highly, while M2 levels increase later in infection, indicating that splicing increases as infection progresses [23]. We were interested in whether this time-dependent increase in the splicing of influenza viral mRNAs would apply to our newly introduced intron in the NA segment. Indeed, we observed increased splicing of the PR8 NA-intron mRNAs over time (**S2A Fig**).

Splicing machinery is generally conserved among vertebrate species; however, splicing is also a recognized host determinant for avian- and mammalian-derived influenza viruses [9–13]. Most notably, avian-adapted influenza viruses have been reported to replicate poorly in mammalian cells due to excessive M splicing [24,25]. Therefore, we were interested in how our NA-intron, which was not specifically adapted to either an avian or mammalian host, would behave in different hosts. We first infected embryonated chicken eggs with WT PR8 and PR8-NA-intron virus and found no observable defect in infectious viral production, suggesting successful viral replication in an avian environment (**Fig 1I**). To more rigorously define potential differences between mammalian and avian growth, we infected human lung epithelial cells (A549) and chicken embryo fibroblast cells (DF-1) with the PR8-NA-intron virus. The replication of PR8-NA-intron was similar in both cell types (**S2B Fig**). However, previous findings show that avian IAV M mRNAs are more frequently spliced in mammalian cells compared to avian cells [24,25] and we similarly observed significantly more splicing of the NA-intron segment in the human A549 cells compared to avian DF-1 cells (**S2C Fig**). These findings indicate that many of the same factors which dictate the canonical splicing of IAV M mRNAs over time and in different hosts likely govern splicing of our artificial intron.

### Artificial introns of different lengths are tolerated and can be engineered to express exogenous proteins

**Fig 2:**
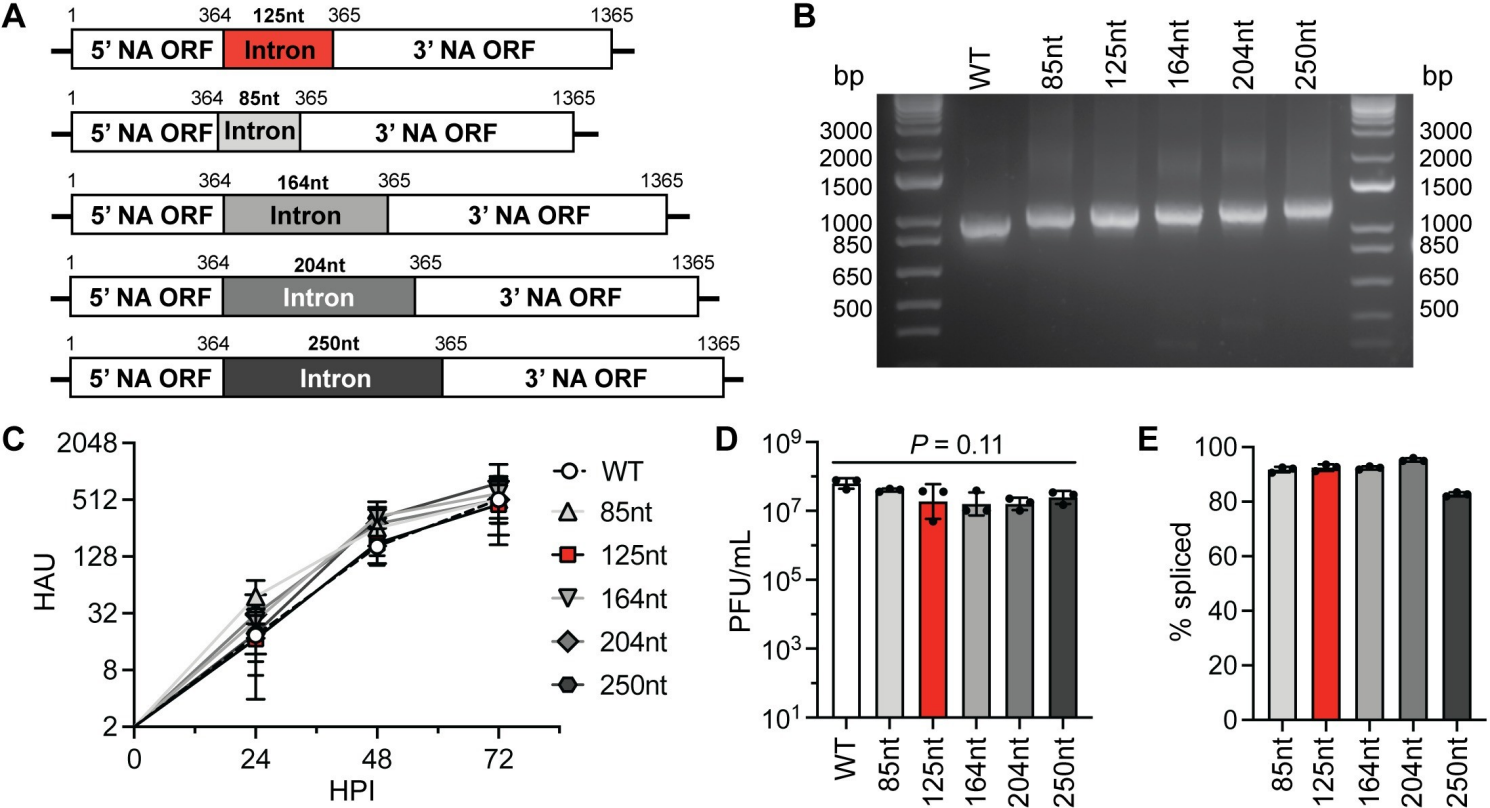
Intronic length has minor effects on viral fitness. (A) Diagrams of PR8 IAV segment 6/NA with artificial introns of varying lengths (gray) compared to the initial sequence (red). (B) RT-PCR for segment 6/NA of WT PR8 virus and PR8-NA-intron (varying lengths) viruses. (C) Growth kinetics of WT PR8 and PR8-NA-intron (varying lengths) virus infections (MOI = 0.001, multicycle) on MDCK cells, measured using HA assays (mean with SD, n = 3 independent experiments). (D) End point titers of WT PR8 and PR8-NA-intron (varying lengths) virus infections (MOI = 0.01, multicycle, 72h) on MDCK cells, measured using plaque assays (mean with SD, n = 3 independent experiments, one-way ANOVA). (E) PR8 segment 6/NA-intron mRNA splicing rates from PR8-NA-intron (varying lengths) virus infections (MOI = 2, single cycle, 8h) on MDCK cells, measured using two-step RT-qPCR (mean with SD, n = 3 independent experiments).

Intron length and cis-elements, both intronic and exonic, are important splicing determinants [26,27]. We therefore wanted to test if the ability of a viral segment to tolerate segment splicing was dependent on the specific characteristics of the intron. As a way to modify the intron itself we first varied the length of the intron, originally 125nt, to 85nt, 164nt, 204nt, or 250nt, in the NA segment and rescued the corresponding viruses in the PR8 background (**Fig 2A and 2B**). Each PR8-NA-intron virus, regardless of intron length, grew to high titers after multicycle infection on MDCK cells (**Fig 2C and 2D**). Additionally, the intron-retained, unspliced mRNA remained the minor product compared to the spliced, functional viral protein encoding mRNA (**Fig 2E**).

**Fig 3:**
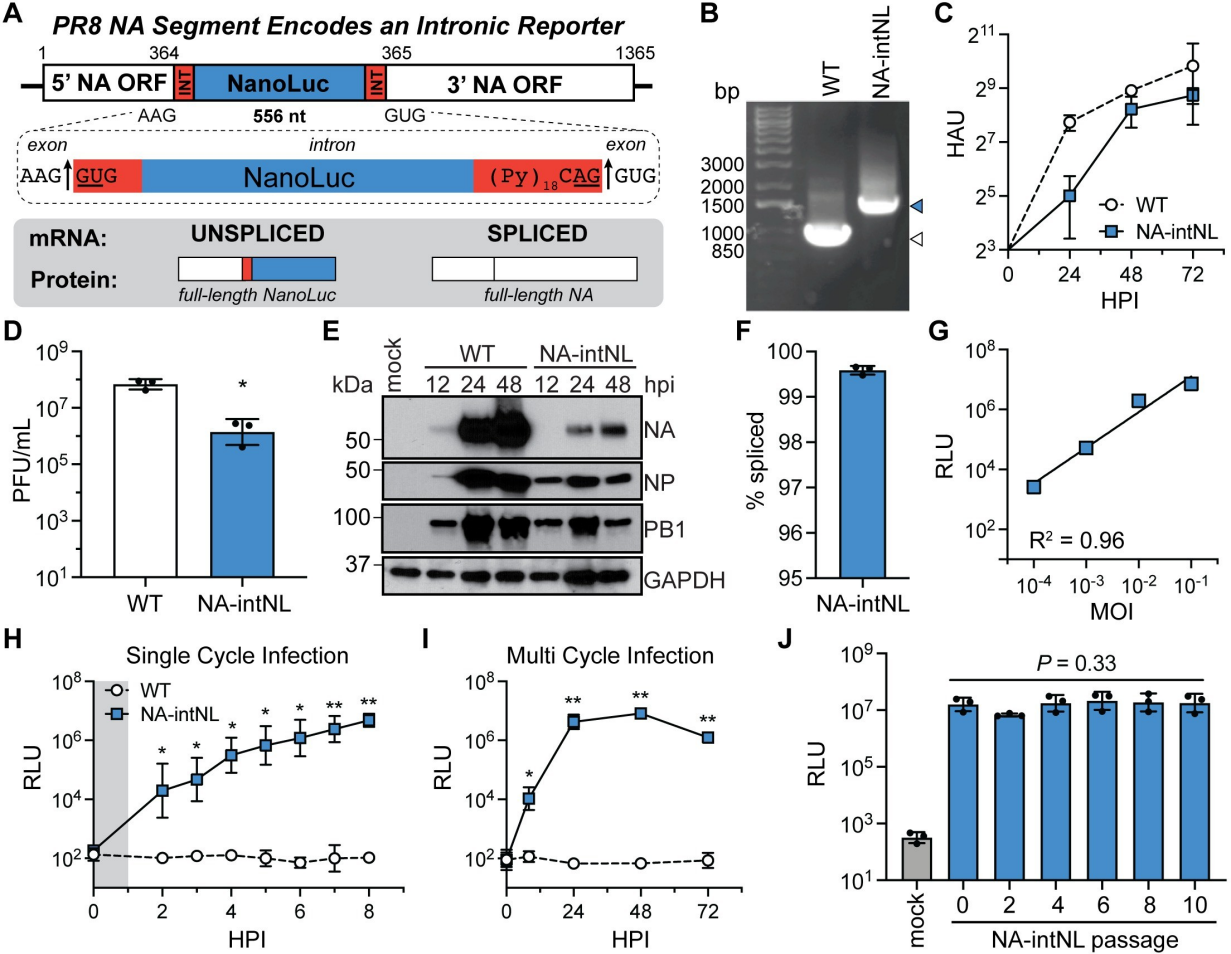
Artificial introns can be used for expression of a reporter protein. (A) Diagram of the PR8 IAV segment 6/NA containing an intron encoding a NanoLuc (NL) reporter and its protein products. (B) RT-PCR of WT PR8 virus (white) and PR8-NA-intNL virus (blue) segment 6/NA. (C) Growth kinetics of WT PR8 and PR8-NA-intNL virus infections (MOI = 0.001, multicycle) on MDCK cells, measured using HA assays (mean with SD, n = 3 independent experiments). (D) End point titers of WT PR8 and PR8-NA-intNL virus infections (MOI = 0.01, multicycle, 72h) on MDCK cells, measured using plaque assays (mean with SD, n = 3 independent experiments, unpaired Student’s t-test). (E) Western blots for PR8 NA, NP and PB1 proteins from mock, PR8 WT, and PR8-NA-intNL virus infections (MOI = 0.01, multicycle) on MDCK cells (representative of three independent experiments). GAPDH was used as a loading control. (F) PR8 segment 6/NA-intNL mRNA splicing rates from PR8-NA-intNL virus infections (MOI = 2, single cycle, 8h) on MDCK cells, measured using two-step RT-qPCR (mean with SD, n = 3 independent experiments). (G) Luciferase levels from PR8-NA-intNL virus infections at different MOIs (single cycle, 8h) on MDCK cells (mean with SD, n = 3 independent experiments, simple linear regression with goodness of fit). MOI, multiplicity of infection; RLU, relative light units. (H) Luciferase levels from WT PR8 and PR8-NA-intNL single cycle virus infections (MOI = 0.1, single cycle) on MDCK cells (mean with SD, n = 3 independent experiments, unpaired Student’s t-test relative to WT). Gray shading indicates infection incubation period. (I) Luciferase levels from WT PR8 and PR8-NA-intNL multicycle virus infections (MOI = 0.001, multicycle) on MDCK cells (mean with SD, n = 3 independent experiments, unpaired Student’s t-test relative to WT). (J) Luciferase levels from infections (MOI = 0.001, multicycle, 24h) on MDCK cells using PR8-NA-intNL virus (passage 0) and supernatants from serial PR8-NA-intNL virus infections (MOI = 0.001, multicycle, 72h passages) on MDCK cells (mean with SD, n = 3 independent experiments, one-way ANOVA). For all panels: *P < 0.05, **P < 0.001 and ns = not significant.

Since our artificial introns were spliced in ~90% of mRNAs (**Fig 1F**), we next wanted to determine if we could use the remaining ~10% of transcripts retaining the intron to expand the coding capacity of the IAV genome. Therefore, we selected the NanoLuc gene as a model ORF for insertion based on its small size (516nt) and detectable activity at low levels of expression [28,29]. We inserted a constitutively spliced, intron-flanked NanoLuc gene in frame at the previous location in the PR8 NA segment such that the unspliced mRNAs would now express the NanoLuc protein (**Fig 3A**). We successfully rescued the virus in the PR8 genetic background and showed that, while the PR8-NA-intNL virus had delayed growth kinetics relative to WT virus, it grew to high, but somewhat reduced, titers (**Fig 3B–3D**). As expected, the reduced kinetics of replication and lower end point titers could also be observed at the viral protein level over time (**Fig 3E**). Unexpectedly, splicing of the NA mRNA increased with the larger, reporter-containing intron, from an ~90% splicing rate in our PR8-NA-intron virus to more than 99% spliced in the PR8-NA-intNL virus (**Fig 3F**). Despite the low production of NanoLuc-containing transcripts, luciferase activity correlated well with infectious dose and high levels of reporter gene expression were detectable during single- and multi-cycle virus infections on MDCK cells (**Fig 3G–3I**). Thus, genes can functionally be expressed from artificial intron-containing influenza viral segments.

For intronic reporter viruses to have practical utility they must be stable throughout an experiment and ideally through multiple rounds of propagation. We therefore expanded our passaging experiments and found that, after 10 passages of the PR8-NA-intNL virus on MDCK cells, luciferase activity remained insignificantly changed from the virus stock and RT-PCR and sequencing of PR8 segment 6 demonstrated that the intronic NanoLuc reporter was stable (**Figs 3J and S3A**). After passaging on human lung A549 cells we observed similar results to the MDCK cell experiments (**S3B and S3C Fig**). We next tested viral growth and stability in embryonated chicken eggs. While the PR8-NA-intNL virus replicated and produced high levels of luciferase in this environment, the intron was sometimes lost after multiple rounds of serial passage (**S4A–S4F Fig**). Thus, mammalian culture methods are preferable for propagating intronic reporter viruses.

### Intron-reporter containing viruses can be used for cell-based screening assays

**Fig 4:**
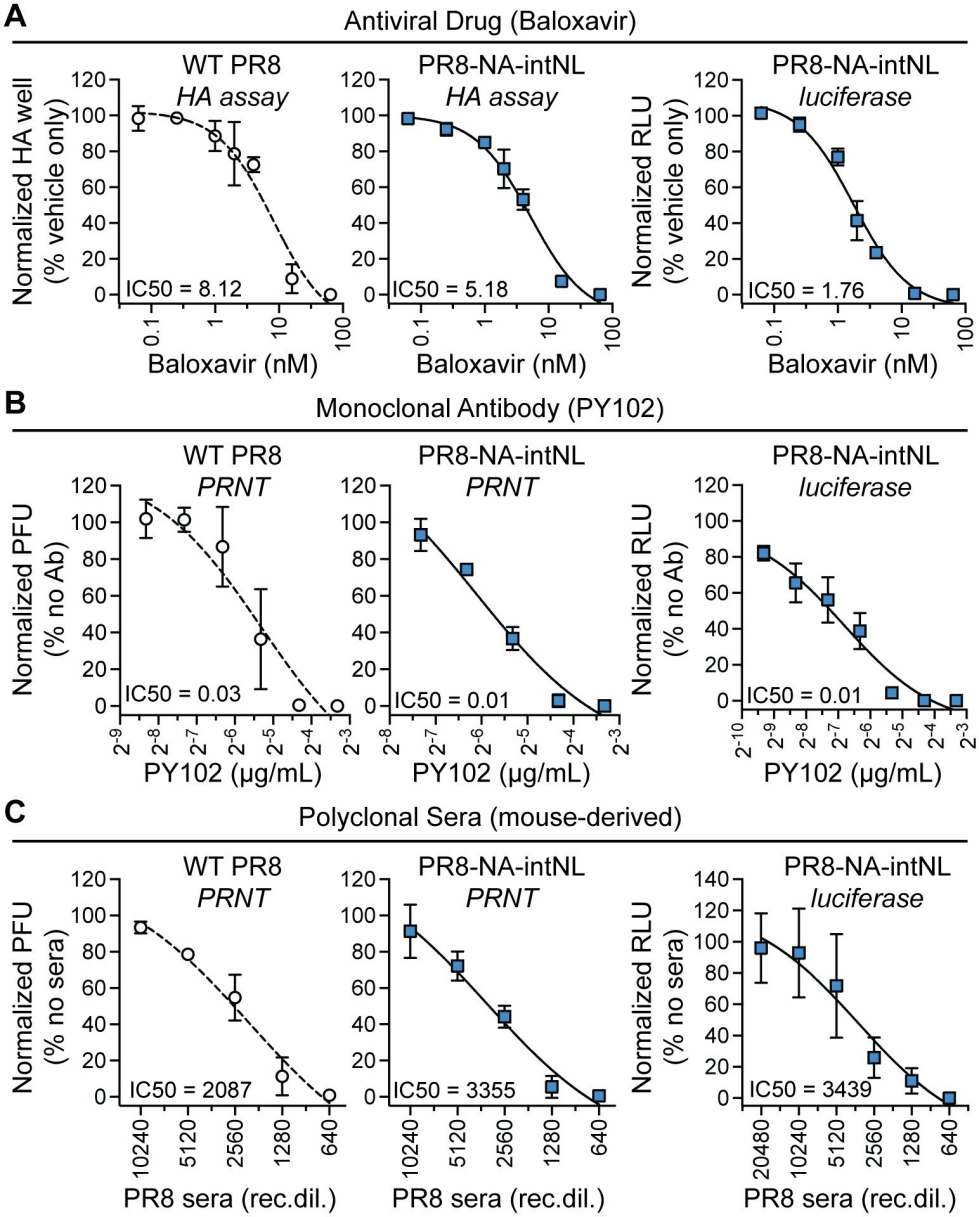
IAVs harboring introns with luciferase genes can be used to evaluate antibodies and antiviral compounds. (A) Virus levels from Baloxavir treated WT PR8 (left) and PR8-NA-intNL (middle) virus infections (MOI = 0.05, multicycle, 48h) measured using HA assays and luciferase levels from Baloxavir treated PR8-NA-intNL (right) virus infections (MOI = 0.01, multicycle, 24h) on MDCK cells (mean with SD, n = 3 independent experiments, nonlinear regression dose-response curve). Normalized to vehicle-treated, infected controls. (B) Virus levels from PY102 anti-H1 antibody pre-incubated WT PR8 (left) and PR8-NA-intNL (middle) virus infections (40 virions/well) measured using PRNTs and luciferase levels from PY102 anti-PR8 HA antibody pre-incubated PR8-NA-intNL (right) virus infections (MOI = 0.001, multicycle, 24h) on MDCK cells (mean with SD, n = 3 independent experiments, nonlinear regression dose-response curve). Normalized to PBS pre-incubated controls. PRNTs, plaque reduction neutralization tests. (C) Virus levels from anti-PR8 mouse sera pre-incubated WT PR8 (left) and PR8-NA-intNL (middle) virus infections (40 virions/well) measured using PRNTs and luciferase levels from anti-PR8 mouse sera pre-incubated PR8-NA-intNL (right) virus infections (MOI = 0.001, multicycle, 24h) on MDCK cells (mean with SD, n = 3 independent experiments, nonlinear regression dose-response curve). Normalized to PBS pre-incubated controls; rec.dil., reciprocal dilution.

Luciferase reporter viruses have previously been utilized in many applications, including as tools for influenza virus antiviral drug, neutralizing antibody, and immune sera screening [30–35]. We next tested our reporter virus in these contexts relative to unmodified, wild-type virus. First, we measured the effect of a recognized influenza antiviral Baloxavir, a cap-dependent endonuclease inhibitor that blocks influenza PA activity [36]. Using a hemagglutination assay readout, we found both viruses were inhibited at similar drug levels (**Fig 4A**). We also collected PR8-NA-intNL virus-infected, Baloxavir-treated cells for luciferase assays and, using luciferase signal, determined a comparable inhibitory concentration (**Fig 4A**). We then performed plaque reduction neutralization tests (PRNTs) using the anti-PR8 neutralizing monoclonal antibody PY102 [37] with WT PR8 and PR8-NA-intNL viruses and found the neutralizing antibody inhibited both viruses with similar IC50s (**Fig 4B**). Decreased reporter activity from MDCK cells infected with PR8-NA-intNL virus that were pre-incubated with PY102 antibody also correlated well with the antibody-based inhibition of infectious virus levels (**Fig 4B**). Analogous experiments using mouse-derived, anti-PR8 polyclonal serum showed our intron-reporter virus is also suitable for neutralizing sera-based experiments (**Fig 4C**). Thus, intronic reporter viruses have utility in many common reporter virus assays.

### An intron-reporter IAV strain replicates and is able to cause disease *in vivo*

**Fig 5:**
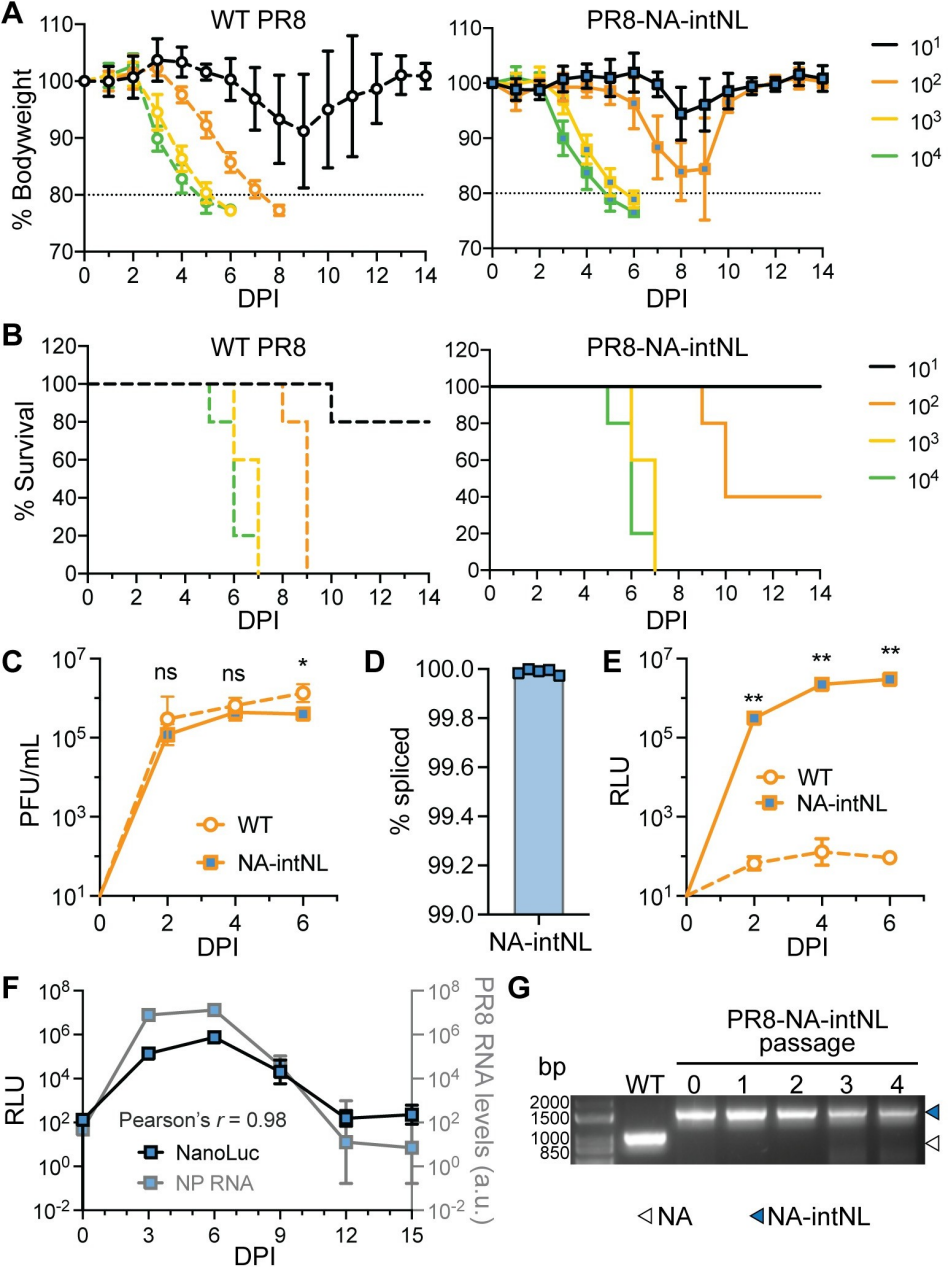
IAVs harboring introns with luciferase genes cause disease in a mouse model. (A) Relative bodyweights of WT PR8 (left) and PR8-NA-intNL (right) virus infected C57BL/6J mice (age matched, 6–12 weeks, female) (mean with SD, n = 5 mice per group). DPI, days post-infection. (B) Survival of WT PR8 (left) and PR8-NA-intNL (right) virus infected BL/6 mice (n = 5 mice per group). (C) Lung viral titers from WT PR8 and PR8-NA-intNL virus infected (100PFU) BL/6 mice, measured using plaque assays (mean with SD, n = 5 mice per group, unpaired Student’s t-test relative to WT). (D) PR8 segment 6/NA-intNL mRNA splicing rates from PR8-NA-intNL virus infected (100PFU, 4d) BL/6 mouse lungs, measured using two-step RT-qPCR (mean with SD, n = 5 mice per group). (E) Luciferase levels from WT PR8 and PR8-NA-intNL virus infected (100PFU) BL/6 mice lungs (mean with SD, n = 5 mice per group, unpaired Student’s t-test relative to WT). (F) Luciferase and PR8 NP RNA levels from PR8-NA-intNL virus infected (10PFU) BL/6 mouse lungs (mean with SD, n = 3 mice per group, Pearson correlation coefficient). Arbitrary units (a.u.) determined relative to 18S. Background levels of detection from uninfected mice are shown at 0DPI. (G) RT-PCR of WT PR8 virus (white) and PR8-NA-intNL virus (blue) segment 6/NA passages 0 to 4 from serial infections (1000PFU, 3d passages) in BL/6 mouse lungs (representative of three independent experiments). For all panels: *P < 0.05, **P < 0.001 and ns = not significant.

Cancer cells are known to alter the cellular splicing environment [38], and most of our previous experiments had been performed in immortalized cancer cell lines. As a result, we were interested in how the inclusion of an intron in an additional viral segment would impact *in vivo* influenza virus infections. We therefore infected immune competent C57BL/6 mice with a range of doses of WT PR8 virus or the PR8-NA-intNL virus and measured their bodyweight loss as an indicator of disease. The PR8-NA-intNL virus resulted in both mouse weight loss and mortality, though at higher viral doses compared to WT PR8 (**Fig 5A and 5B**). Lung virus growth kinetics were similar between WT PR8 and PR8-NA-intNL virus infections at a potentially lethal viral dose (100 PFU) (**Fig 5C**). Despite a high degree of intron-reporter splicing (greater than 99% spliced) (**Fig 5D**), we detected luciferase activity from mouse lung homogenates following the pattern expected from viral growth kinetics (**Fig 5E**). We also infected mice with a sublethal dose of the PR8-NA-intNL virus (10 PFU) and observed a strong correlation (r = 0.98) between luciferase activity and viral RNA at all timepoints (**Fig 5F**). Finally, we serially passaged the PR8-NA-intNL virus in mouse lungs and found the NanoLuc reporter remained stable (**Figs 5G and S5A–S5D**).

### An intron-encoded reporter gene is also tolerated in the IAV NP segment

**Fig 6:**
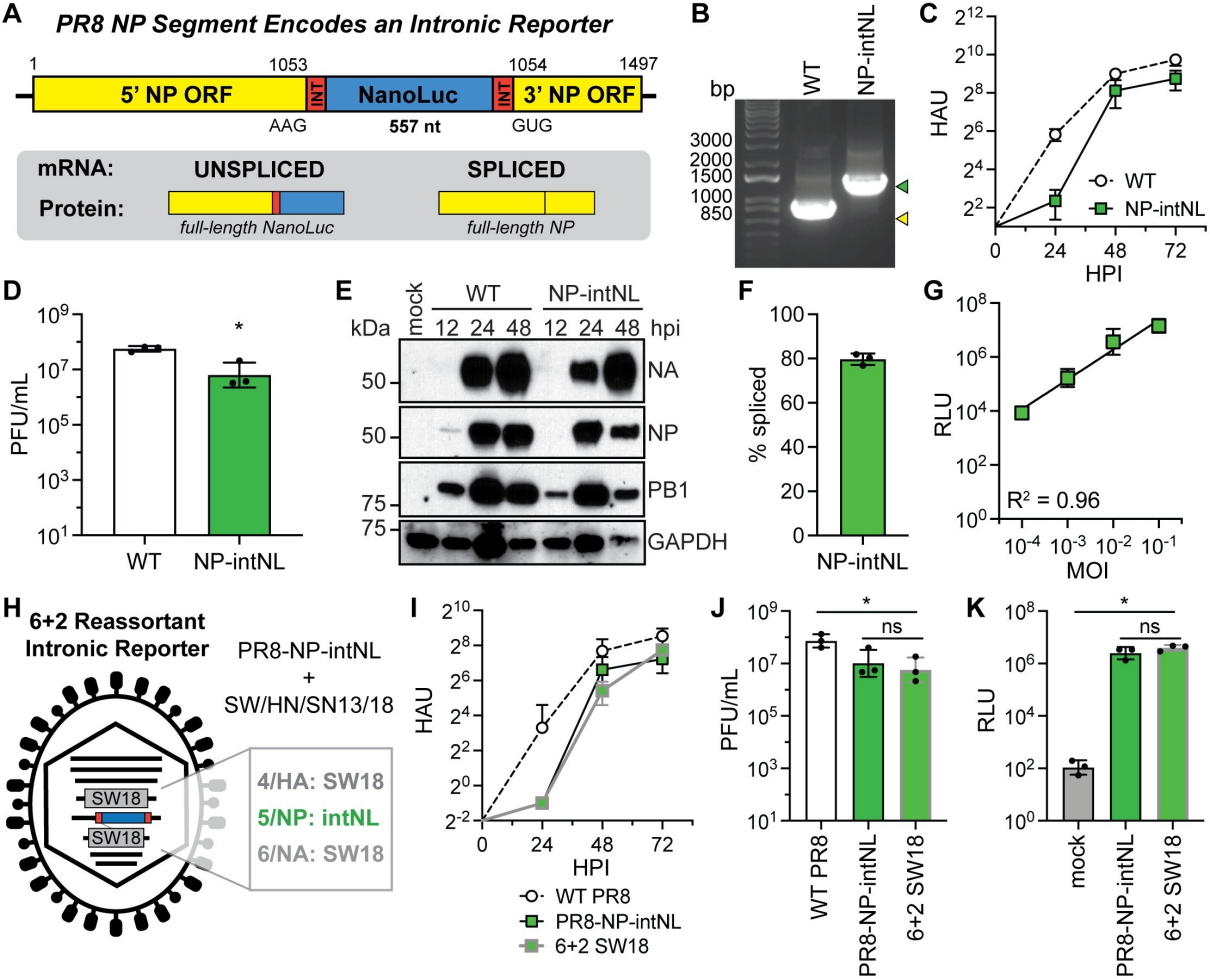
Intronic reporters in PR8 segment 5 allow generation of 6+2 reassortment viral reporter strains. (A) Diagram of the PR8 IAV segment 5/NP containing an intron encoding a NanoLuc reporter and its protein products. (B) RT-PCR of WT PR8 virus (yellow) and PR8-NP-intNL virus (green) segment 5/NP. (C) Growth kinetics of WT PR8 and PR8-NP-intNL virus infections (MOI = 0.001, multicycle) on MDCK cells, measured using HA assays (mean with SD, n = 3 independent experiments). (D) End point titers of WT PR8 and PR8-NP-intNL virus infections (MOI = 0.01, multicycle, 72h) on MDCK cells, measured using plaque assays (mean with SD, n = 3 independent experiments, unpaired Student’s t-test). (E) Western blots for PR8 NA, NP and PB1 proteins from mock, PR8 WT, and PR8-NP-intNL virus infections (MOI = 0.01, multicycle) on MDCK cells (representative of three independent experiments). GAPDH was used as a loading control. (F) PR8 segment 5/NP-intNL mRNA splicing rates from PR8-NP-intNL virus infections (MOI = 2, single cycle, 8h) on MDCK cells, measured using two-step RT-qPCR (mean with SD, n = 3 independent experiments). (G) Luciferase levels from PR8-NP-intNL virus infections at different MOIs (single cycle, 8h) on MDCK cells (mean with SD, n = 3 independent experiments, simple linear regression with goodness of fit). (H) Diagram of the 6+2 influenza reassortant with SW/HN/SN13/18 (SW18) glycoproteins on a PR8-NP-intNL background. (I) Growth kinetics of WT PR8, PR8-NP-intNL, and 6+2 SW18 virus infections (MOI = 0.001, multicycle) on MDCK cells, measured using HA assays (mean with SD, n = 3 independent experiments). The downward error bar for the WT PR8 24h data point could not be plotted on a log scale. (J) End point titers of WT PR8, PR8-NP-intNL, and 6+2 SW18 virus infections (MOI = 0.001, multicycle, 72h) on MDCK cells, measured using plaque assays (mean with SD, n = 3 independent experiments, unpaired Student’s t-test). (K) Luciferase levels from mock, PR8-NP-intNL, and 6+2 SW18 virus infections (MOI = 0.001, 24h, multicycle) on MDCK cells (mean with SD, n = 3 independent experiments, unpaired Student’s t-test). For all panels: *P < 0.05, **P < 0.001 and ns = not significant.

We were next interested to see if an artificial NanoLuc encoding intron inserted into a different viral genomic locus would be viable and if the resulting virus would have similar characteristics to the NA-intNL virus. We therefore incorporated the intron-sequence-flanked NanoLuc reporter into segment 5, which encodes the NP protein, using the same insertion scheme as for segment 6 (**Fig 6A**). Indeed, we were able to rescue a PR8-NP-intNL virus which grew to high titers, though with delayed kinetics and ultimately reduced titers relative to WT virus, on MDCK cells (**Fig 6B–6E**). In contrast to the NA-intNL mRNA, the NP-intNL mRNA only spliced ~80% of the time (**Figs 6F and S6A**). High luciferase signal was detected after infection, and the luciferase levels correlated well with viral MOI (**Fig 6G**). In order to define the stability of the reporter at this viral locus, we performed serial passaging on MDCK cells. In contrast to the NA-intNL virus, we observed a loss in reporter signal, segment length and NanoLuc sequence after several rounds of serial passage (**S6B Fig**). Thus, not all intronic insertion sites produce viruses with the same characteristics, and some of these genomic modifications impart a significant defect on viral fitness.

One potential benefit of utilizing a non-glycoprotein encoding intron insertion site such as segment 5 is that segments 4 and 6 can be exchanged with corresponding segments from other strains. These so-called “6+2” reassortants (harboring internal segments from a laboratory adapted strain such as PR8 and the glycoprotein segments from a contemporary strain) are frequently generated to improve vaccine yields or to facilitate growth in animal models of infection [39]. To show that PR8 NP segments harboring reporter introns have utility for this approach, we generated a virus with the glycoproteins from the recently characterized H1N1 G4 swine virus A/swine/Henan/SN13/2018 (SW/HN/SN13/18, SW18) [40] along with the 6 remaining segments from PR8 (**Fig 6H**). The G4/PR8-NP-intNL virus grew to high titers and produced similar luciferase activity levels compared to PR8-NP-intNL virus (**Fig 6I–6K**), demonstrating the potential utility of this approach.

### Intron-based reporters are a generalizable approach for the development of reporter influenza virus strains

**Fig 7:**
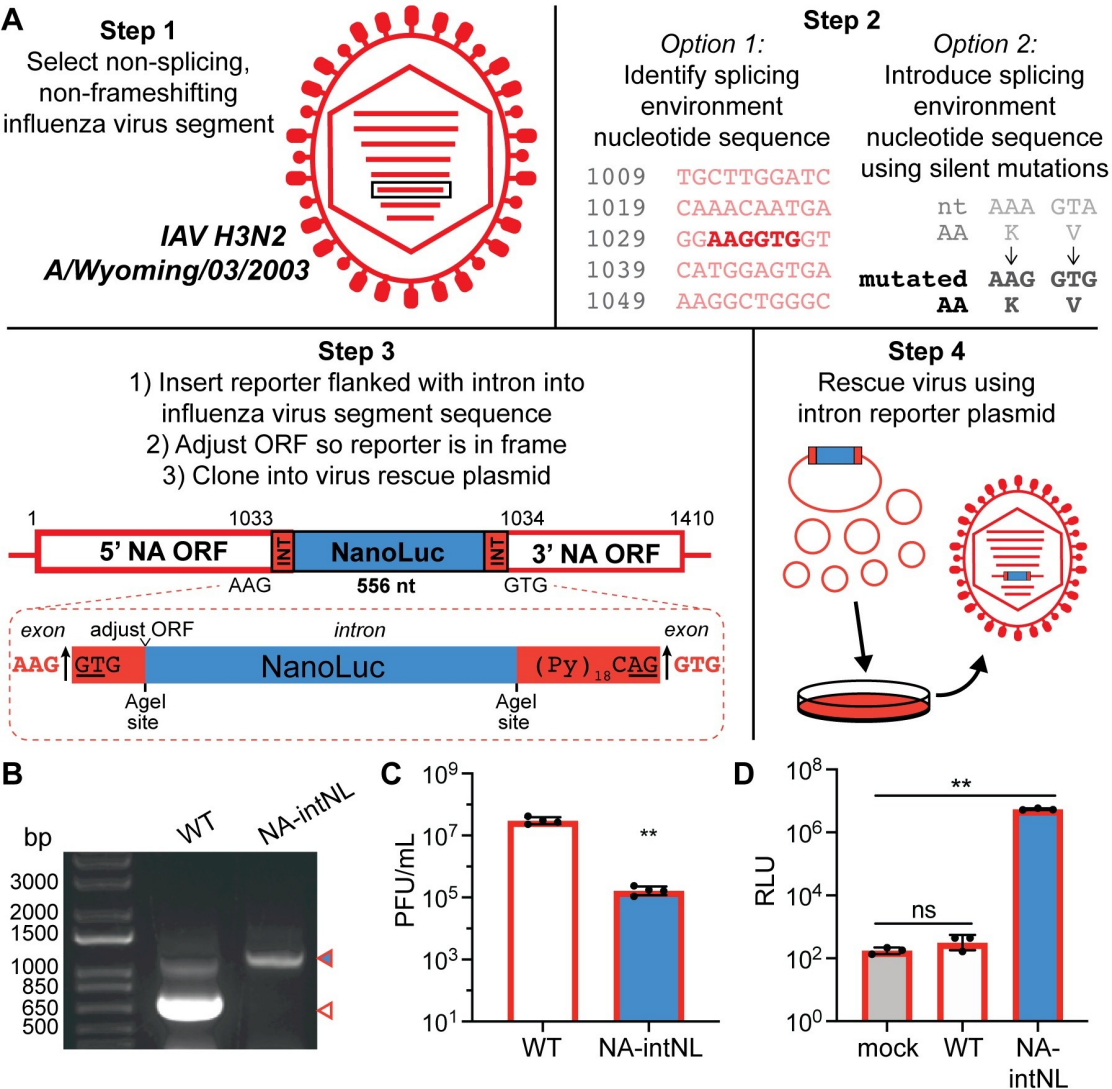
Artificial intron insertion can be used to develop reporter strains in different viral genetic backgrounds. (A) Diagram demonstrating how to introduce an artificial intron encoding a reporter into non-splicing, non-frameshifting IAV segments using A/Wyoming/03/2003 (H3N2) segment 6/NA as an example. (B) RT-PCR of WT Wyo/03 virus (white) and Wyo/03-NA-intNL virus (blue) segment 6/NA. (C) End point titers of WT Wyo/03 and Wyo/03-NA-intNL virus infections (MOI = 0.0001, multicycle, 72h) on MDCK cells, measured using plaque assays (mean with SD, n = 4 independent experiments, unpaired Student’s t-test). (D) Luciferase levels from WT Wyo/03 and Wyo/03-NA-intNL virus infections (MOI = 0.0001, multicycle, 24h) on MDCK cells (mean with SD, n = 3 independent experiments, unpaired Student’s t-test). For all panels: *P < 0.05, **P < 0.001 and ns = not significant.

Finally, we were interested in testing if our newfound knowledge regarding IAV tolerance of artificial introns could be leveraged as a generalizable platform to generate reporter influenza viruses. We therefore selected an H3N2 IAV, A/Wyoming/03/2003 (Wyo/03) that is highly divergent from PR8. We then developed a set of design guidelines based on all of the data we had previously generated (**Fig 7A**). First, we recommend selecting a normally nonsplicing segment as well as one without multiple overlapping reading frames to theoretically maximize productive reporter translation. We selected the Wyo/03 NA segment because while that protein is of a different subtype and unrelated to the the PR8 NA, the NP segment is reasonably conserved between the two viruses. In our case, we identified the nucleotide sequence “AAGGUG” in the Wyo/03 NA ORF; however, if a viral segment does not contain the nucleotide sequence “AAGGUG,” it may be introduced using silent mutations (**S1 Table**). We then inserted the intron-flanked NanoLuc sequence between the “AAG” and “GUG” nucleotides and verified that NanoLuc was in the correct reading frame. Finally, we rescued the Wyo/03-NA-intNL virus on MDCK cells (**Fig 7B**). The Wyo/03-NA-intNL virus grew to lower titers compared to WT Wyo/03 H3N2 virus (**Figs 7C and S7**). However, we detected significant luciferase activity during Wyo/03-NA-intNL virus infection of MDCK cells demonstrating the successful translation of the unspliced reporter-encoding Wyo/03 NA-intNL mRNA (**Fig 7D**). Thus, including an intron reporter sequence in a normally intronless IAV segment is a viable, and likely broadly generalizable, method for producing novel reporter viruses.

## Discussion

Influenza viruses take advantage of host splicing machinery to produce multiple functional proteins from a single viral segment. In this paper, we explored the constraints on IAV genomic splicing and leveraged our findings to generate IAV reporter strains by introducing intronic reporters into otherwise nonsplicing viral segments. Overall, this work demonstrates that adapting a viral method of host hijacking, specifically taking advantage of the host splicing machinery, and applying it to additional segments is both a permissible and practical method for expanding the coding capacity of influenza viruses.

Nevertheless, there are a number of questions that remain unanswered. First of all, we found that our introduced artificial introns were spliced at different rates depending on the intronic and exonic sequences; however, it remains unclear exactly why this is the case. These differences are likely at least partially the results of mRNA cis-elements that help govern splicing. For example, an exonic splicing enhancer in the 3’ M segment recruits the SF2 splicing factor to stabilize splicing, contributing to the balance between M1, M2, and mRNA3 transcripts [41]. Further, splicing rates are also impacted by secondary structures in the RNA [42–45]. None of these variables were accounted for in our designs, but understanding how they affect splicing will likely refine our ability to control the frequency of removal or retention of artificial introns and improve the effectiveness of the intron-based reporter viruses.

In addition to the cis-elements, the cellular environment also impacts how splicing occurs. From our experiments with human/A549 vs. avian/DF-1 cells and MDCK cells over time, we found that our newly splicing segment was impacted by these different environments (**S2A–S2C Fig**). However, influenza virus infection is an additional environment in itself. It has been proposed that influenza NS1 represses splicing of both viral transcripts (as a way to increase NS1 transcripts) and host transcripts (as a mechanism of host shutoff) [46]. Similarly, it is thought that the viral polymerase complex helps direct splicing to the weaker splice site of M2 over the stronger splice site of mRNA3 to increase production of M2 [47]. Future studies will be required to understand how these (and potentially other) characteristics of the viral RNA itself or the cellular environment during infection affect the splicing of artificial introns.

We also demonstrated how artificial introns can be used for the generation of novel influenza reporter viruses. One advantage of this approach over previous luciferase reporter influenza viruses is that RNA packaging signal mapping and/or manipulation is not required [48]. Furthermore, it may be possible to introduce introns into multiple segments and produce multiple reporter proteins at the same time. Another benefit of the system is its flexibility. We have already shown that PR8 segments NA and NP tolerate the intronic reporters. Using the Influenza Research Database, we found among searchable IAV NA and NP sequences, greater than 100,000 segments contained amino acid sequences compatible with introducing the “AAGGUG” nucleotide sequences [49]. This analysis demonstrates our design guidelines for incorporating intronic reporters to produce novel reporter influenza viruses are widely applicable.

However, there are additional considerations when generating a reporter virus by inserting an intron. First, as currently constructed, the reporter is fused to the 5’ sequence of the viral gene ORF; for our NA-intNL viruses NanoLuc is fused with the stalk domain and potentially being trafficked to the membrane and incorporated into the virion, likely reducing viral fitness. To prevent this fusion, a 2A cleavage-site could be incorporated ahead of the reporter reading frame. Another aspect of consideration is codon usage as introns are partially identified by their different GC content compared to their adjacent exons [50]. Since IAVs have low GC content relative to their hosts [51,52], it is potentially important to consider how the primary sequence of the intron relates to the viral background. Overall, with these considerations in mind, we believe inserting intronic reporters into intronless viral segments is a promising, generalizable way to generate new influenza reporter viruses.

In sum, we sought to learn whether additional IAV segments could tolerate splicing; by experimental introduction of artificial introns, we found that not only were introns tolerated, but they could be used to express additional proteins. While we leveraged these observations to generate viral reporter strains, the approaches described in this work represent new tools that may be able to aid in understanding the mechanisms that normally underly splicing in the influenza genome. Future rational use of artificial introns to modify influenza viral genomes has broad utility and will facilitate investigation into molecular virology, viral pathogenesis, and translational research questions.

## Materials and methods

### Ethics statement

Animal procedures were performed in compliance with the Duke University IACUC approved protocols A189-18-08 and A142-21-07. The Duke University animal program is registered with the United Stated Department of Agriculture Animal Welfare Act (#863), approved through the National Institutes of Health Policy on Humane Care and Use of Laboratory Animals (#D16-00123, A3195-01), and accredited by AAALAC International (#363). Animals were assessed daily for signs of distress (change in respiratory rate, reduced movement, ruffled fur, change in grooming behaviors, agitation, lethargy) and bodyweight loss. Bodyweight loss of 20% compared to starting weight was the primary determinant of humane endpoints. CO_2_ asphyxiation was used for primary euthanasia with bilateral thoracotomy as a secondary method.

### Cell culture

Cells were obtained from ATCC and grown at 37°C in 5% CO_2_. Madin-Darby canine kidney (MDCK) cells were grown in minimal essential medium (MEM) with 5% fetal bovine serum (FBS), GlutaMax, HEPES, NaHCO_3_, and penicillin-streptomycin. Human alveolar basal adenocarcinoma epithelial (A549) and chicken embryo fibroblasts (DF-1) cells were grown in Dulbecco’s modified Eagle’s medium (DMEM) with 10% FBS, GlutaMax, and penicillin-streptomycin. Human embryonic kidney 293T cells were grown in Dulbecco’s modified Eagle’s medium (DMEM) with 5% FBS, GlutaMax, and penicillin-streptomycin. All cells were maintained in plasmocin (2.5μg/mL).

### Viruses

Recombinant viruses were generated by first inserting the desired segment with an intron sequence (**S8 and S9 Figs**) into the pDZ vector using platinum *Taq* PCR (Invitrogen) and the NEBuilder HiFi DNA assembly kit (New England BioLabs). A/swine/Henan/SN13/2018 HA (GenBank: MN416622) and NA (GenBank: MN416726) ORF sequences were cloned into PR8 packaging signals for their corresponding segment. DNA with the desired sequences were synthesized by Integrated DNA Technologies, Inc. The recombinant plasmids were transfected into low passage 293T cells with the WT versions of the remaining segments in pDZ plasmids using Trans-IT LT1 transfection reagent (Mirus). Supernatant was collected after 48 hours after transfection and used to infect MDCK cells. Supernatant from MDCK cells was collected 48–72 hours later and further used to infect MDCK cells or embryonated chicken eggs for plaque purification of rescued viruses. Unmodified A/Puerto Rico/8/1934 (PR8) and A/Wyoming/03/2003 (Wyo/03) viruses were grown and propagated in chicken eggs or on MDCK cells. Modified segments of viruses used in this study were reverse-transcribed and sequenced via Sanger sequencing.

### Cell culture infections

Cells were washed with PBS before being infected with virus diluted in PBS/BSA infection media. Cells were infected for 45 minutes and agitated every 10 minutes. Infection media was then removed and replaced with complete media for single cycle infections or post-infection media supplemented with TPCK trypsin for multicycle infections depending on experimental design.

### Hemagglutination (HA) assays

Cell supernatant containing virus was diluted with cold PBS 1:2 for at least 8 dilutions in a V-bottom 96-well plate. 50 μl of cold PBS containing a 1:40 dilution of chicken or turkey blood was added to the diluted virus wells, and the plate gently swirled to mix. Assays were incubated at 4°C for at least 30 minutes before analysis. HA units were defined as the reciprocal of the highest dilution where hemagglutination was observed.

### Plaque assays

MDCK cells were washed with phosphate-buffered saline (PBS) then infected with 1:10 serially diluted virus for 45 minutes before virus was removed and replaced with an agar overlay. Cells were incubated at 37°C for 48hours before being fixed with 4% paraformaldehyde (PFA) in PBS for at least 3 hours. The agar overlay was then removed, and plaques were incubated overnight at 4°C in sera or antibody diluted in antibody dilution buffer (5% nonfat dried milk, 0.05% Tween 20 in PBS). For viruses with PR8 glycoproteins anti-PR8 sera (derived from WT PR8 infected or immunized mice) was used; for 6+2 SW18 reassortant virus the anti-H1 stalk antibody 6F12 (mouse, kind gift from Peter Palese) was used; for Wyo/03 viruses the anti-H3 antibody 9H10 (mouse, kind gift from Peter Palese) in combination with anti-X-31 sera (derived from X-31 infected or immunized mice) was used. Plaques were washed with PBS and then incubated for 1 hour in anti-mouse IgG horseradish peroxidase (HRP)-conjugated sheep (GE Healthcare) diluted in antibody dilution buffer. Plaques were washed with PBS and then stained with a TrueBlue peroxidase substrate (KPL) before being air-dried and counted.

### RT-PCR

Viral stocks, infection supernatants, infected egg allantoic fluid, or infected mouse lung homogenates were combined with Trizol (Ambion) and RNA was isolated and resuspended in nuclease-free water. Isolated RNA was reverse transcribed and amplified using SuperScript III One-Step RT-PCR System with Platinum *Taq* High Fidelity DNA Polymerase (Invitrogen) using primers targeting either the 5’ or 3’ region (900-1600bp) of the segment of interest. RT-PCR samples were run on a 1% or 1.5% UltraPure agarose (Invitrogen) gel with SYBR Safe (Invitrogen) and imaged.

### Embryonated chicken egg infections

10-day old chicken eggs were injected in the allantois with 100ul virus diluted in PBS. The injection sites were sealed with wax and infected eggs were maintained at 37°C until the designated collection time when eggs were moved to 4°C overnight. Once eggs were completely cooled, the virus-containing allantoic fluid was collected.

### RNA preparations for RT-qPCR

RNA samples from cell culture were prepared using the Monarch Total RNA Miniprep Kit (New England BioLabs). RNA samples from mouse lung homogenates were collected in Trizol (Ambion) and prepared according to the Phasemaker Tube protocol (Invitrogen).

### One-step, probe-based RT-qPCR

RNA samples were analyzed using the EXPRESS Superscript One-Step qRT-PCR kit (Thermo Fisher) with primer/probes targeting the PR8 NA and NP RNAs (**S2 Table**) (IDT) and eukaryotic 18S rRNA (Applied Biosystems) on an Applied Biosystems QuantStudio3 instrument.

### Two-step, dye-based RT-qPCR

RNA samples were converted to cDNA with the PrimeScript RT reagent Kit (Perfect Real Time) (Takara) using only the included Oligo dT Primer. cDNA samples were analyzed using SsoAdvanced Universal SYBR Green Supermix (Bio-Rad) with unspliced/spliced isoform-specific primers targeting PR8 M and NS mRNAs and recombinant PR8 NA-intron/intNL and NP-intNL mRNAs (**S3 Table**) on an Applied Biosystems QuantStudio3 instrument.

### Western blotting

Protein samples were collected via chemical cell lysis using RIPA buffer (10 mM Tris-HCl pH 7.5, 1 mM EDTA pH 8.0, 1% Triton X-100, 0.1% sodium deoxycholate, 140 mM NaCl, 0.1% SDS) and normalized by total protein concentration before adding SDS-PAGE sample buffer (Bio-Rad). Protein samples were loaded and run on a 4–20% polyacrylamide gels (Bio-Rad). Gels were transferred to nitrocellulose membranes before being blocked with PBS containing 5% (w/v) non-fat dried milk and 0.1% Tween-20 for at least 1 hour at room temperature or overnight at 4°C. Membranes were incubated with primary antibody diluted in PBS containing 5% (w/v) non-fat dried milk and 0.1% Tween-20 for at least 1 hour at room temperature or overnight at 4°C overnight. Primary antibodies used included anti-N1 (4A5, gift from Gene Tan at J. Craig Venter Institute), anti-NP (GeneTex GTX125989), anti-PB1 (GeneTex GTX125923), and anti-GAPDH (Abcam ab181603). Membranes were washed 3 times with PBS containing 0.1% Tween-20 before being incubated with anti-mouse-HRP (Invitrogen A16072) or anti-rabbit-HRP (Invitrogen A16104) secondary antibodies for 1 hour at room temperature. Membranes were washed 3 times with PBS containing 0.1% Tween-20 before treatment with Clarity or Clarity Max ECL (Bio-Rad) and exposure to film for development. Uncropped Western blots are shown in **S10 Fig**.

### Luciferase assays

Infected cells were lysed in 1x Luciferase Cell Lysis Reagent (Promega) while shaking at room temperature for 20 minutes then moved to a 96-well V-bottom plate. Settled samples were moved to luminometer tubes. Nano-Glo Luciferase Assay Kit (Promega) reagents were prepared and combined with lysed cells, egg allantoic fluid, or mouse lung homogenates for a standard amount of time before being read using an EG&G Berthold Lumat LB 9507 machine. If samples read overload, all samples in that experiment were diluted 1:10 and reread and their reported values were multiplied by 10 to reflect the dilution factor.

### Plaque reduction neutralization tests (PRNTs)

Viruses were incubated with PY102 antibody (mouse anti-PR8 HA, provided by Tom Moran at the Experimental Therapeutics Institute at the Icahn School of Medicine at Mount Sinai) or anti-PR8 sera (derived from WT PR8 infected or immunized mice) dilutions in PBS/BSA for 45 minutes. MDCK cells were washed with PBS then infected with the virus/antibody or virus/sera dilutions for 45 minutes before virus was removed and replaced with an agar overlay supplemented with TPCK trypsin. The infected plates then incubated for 48 hours at 37°C before being fixed with 4% paraformaldehyde (PFA) in PBS for at least 3 hours. The agar overlay was then removed, and plaques were incubated overnight at 4°C with anti-PR8 sera (derived from WT PR8 infected or immunized mice) diluted in antibody dilution buffer (5% nonfat dried milk, 0.05% Tween 20 in PBS). Plaques were washed with PBS and then incubated for 1 hour in anti-mouse IgG horseradish peroxidase (HRP)-conjugated sheep (GE Healthcare) diluted in antibody dilution buffer. Plaques were washed with PBS and then stained with a TrueBlue peroxidase substrate (KPL) before being airdried and counted.

### Animal infections

6- to 12-week-old age matched BL/6 female mice from Jackson Laboratories were anaesthetized using an injection of ketamine/xylazine. Tails were marked and mice were weighed before being intranasally infected with 40 μl virus diluted in pharmaceutical grade PBS. Mice were weighed daily and euthanized if their body weight reached less than 80% of their starting weight. All procedures were completed according to Duke University IACUC.

### Data analysis and presentation

For all experiments, the statistical analyses used to compare experimental groups are indicated in the corresponding figure legends and were performed using GraphPad Prism. All graphs include data from (and statistical analyses were performed on) 3 independents experiments or ≥3 independent biological entities for egg- and mouse-derived data. Western blots and RT-PCR gel images (of passaged virus experiments) shown are representative of three independent experiments. In cases where values were undetermined or below the limit of detection, statistical analyses were performed using only the detected values–if no values were detected for a given datapoint, it is indicated as not detected (ND) within the graph. In some cases where a viral time course is shown, no pre-infection (0h) experimental samples were collected and the line connecting datapoints simply starts at the graph origin. Data displayed on a log10 scale was log transformed, plotted and analyzed as linear data, and graphed on an power of 10 axis. Data displayed on a log2 scale was plotted, analyzed, and graphed on a log2 axis.

## Supporting information

S1 Fig A two-step RT-qPCR assay to determine PR8 mRNA splicing rates. (A) Diagram of two-step RT-qPCR used to determine splicing rates. First, RNA was collected from infected cells/tissue and reverse-trancribed into DNA using oligo(dT) primers to select for mRNAs. Next, SYBR Green-based qPCR was performed using primers that targeted either 1) all mRNAs derived from an IAV segment, or 2) specifically unspliced mRNAs from that segment. Absolute values for the mRNA transcript copy numbers were determined using a standard curve of known plasmid concentrations encoding the segment of interest. Finally, using the generated standard curve, transcript copy numbers were determined for both 1) all mRNAs and 2) unspliced (un) mRNAs derived from the segment of interest and used to determine what percent of all transcripts from one segment were spliced. (B) Top: Diagram of “M1/2” (striped) and “M1 only” (solid) dye-based qPCR primer locations on PR8 M mRNAs. Bottom, left: Absolute standard curve detecting “M1/2” and “M1 only” sequences from a plasmid containing the PR8 M segment. Bottom, right: Absolute standard curve detecting “M1/2” and “M1 only” sequences from a plasmid containing the PR8 M2 ORF. UTR, untranslated region. (C) Top: Diagram of “NS1/NEP” (striped) and “NS1 only” (solid) dye-based qPCR primer locations on PR8 NS mRNAs. Bottom, right: Absolute standard curve detecting “NS1/NEP” and “NS1 only” sequences from a plasmid containing the PR8 NS segment. Bottom, left: Absolute standard curve detecting “NS1/NEP” and “NS1 only” sequences from a plasmid containing the PR8 NEP ORF. (D) Top: Diagram of “NA-int/NA” (white) and “NA-int only” (red) dye-based qPCR primer locations on PR8 NA-intron mRNAs. Bottom, left: Absolute standard curve detecting “NA-int/NA” and “NA-int only” sequences from a plasmid containing the PR8 NA intron-containing segment. Bottom, right: Absolute standard curve detecting “NA-int/NA” and “NA-int only” sequences from a plasmid containing the PR8 NA segment.
(TIF)

S2 Fig Artificial intron splicing rates are dependent on cellular environments. (A) PR8 segment 6/NA-intron mRNA splicing rates over time from PR8-NA-intron virus infections (MOI = 2, single cycle) on MDCK cells, measured using two-step RT-qPCR (mean with SD, n = 3 independent experiments). (B) PR8 NP RNA levels from PR8-NA-intron virus infections (MOI = 2, single cycle, 8h) on human lung A549 and avian embryonic DF-1 cells, measured using one-step RT-qPCR (mean with SD, n = 3 independent experiments, unpaired Student’s t-test). Arbitrary units (a.u.) determined relative to 18S. (C) PR8 segment 6/NA-intron mRNA splicing rates during PR8-NA-intron virus infections (MOI = 2, single cycle, 8h) on human lung A549 and avian embryonic DF-1 cells, measured using two-step RT-qPCR (mean with SD, n = 3 independent experiments, unpaired Student’s t-test). For all panels: *P < 0.05, **P < 0.001 and ns = not significant.
(TIF)

S3 Fig An intronic reporter sequence in PR8 segment 6 is maintained during virus propagation in cell culture. (A) RT-PCR of WT PR8 virus (white) and PR8-NA-intNL virus (blue) segment 6/NA passages 0 to 10 from serial PR8-NA-intNL virus infections (MOI = 0.001, multicycle, 72h passages) on MDCK cells (representative of three independent experiments). ^#^After passage 10, viruses from infection supernatants were plaque purified and the intron-containing viral genomic segment was sequenced via Sanger sequencing. In all cases the intron and NanoLuc gene were present; however, within the artificial intron we detected ≤3 nucleotide deletions or mismatches in the 3’ region of the intron at the ends of homopolymeric runs. This could either be the result of selection for mutant intron sequences or limitations of the sequencing itself. (B) Diagram of A549 cell passaging experiments. (C) Top: Luciferase levels from infections (multicycle, 24h) on A549 cells using PR8-NA-intNL virus (passage 0) and supernatants from serial PR8-NA-intNL virus infections (MOI = 0.5, multicycle, 72h passages) on A549 cells (mean with SD, n = 3 independent experiments). Bottom: RT-PCR of WT PR8 virus (white) and PR8-NA-intNL virus (blue) virus from passages 0 to 4 from serial PR8-NA-intNL virus infections (MOI = 0.5, multicycle, 72h passages) on A549 cells (representative of three independent experiments).
(TIF)

S4 Fig Embryonated chicken eggs are not optimal for repeated propagation of intronic reporter influenza viruses. (A) Growth kinetics of WT PR8 and PR8-NA-intNL virus infections (100PFU) in embryonated chicken eggs, measured using plaque assays (mean with SD, n = 3 eggs per group, unpaired Student’s t-test relative to WT). (B) Luciferase levels in egg allantoic fluid from WT PR8 and PR8-NA-intNL virus infections (100PFU) in embryonated chicken eggs (mean with SD, n = 3 eggs per group, unpaired Student’s t-test relative to WT). (C) Diagram of egg passaging experiments. (D) Titers in egg allantoic fluid from serial PR8-NA-intNL virus infections (100PFU, 72h passages) in embryonated chicken eggs, measured using plaque assays (mean with SD, n = 3 eggs per group). (E) Luciferase levels in egg allantoic fluid from serial PR8-NA-intNL virus infections (100PFU, 72h passages) in embryonated chicken eggs (mean with SD, n = 3 eggs per group); un, uninfected. (F) RT-PCR of WT PR8 virus (white) and PR8-NA-intNL virus (blue) segment 6/NA from egg passages 0 to 4; exp, independent experiment. ^#^After passage 4, viruses from infected egg allantoic fluid were plaque purified and the intron-containing viral genomic segment was sequenced via Sanger sequencing. In all cases we detected a mixed population within one stock, with some apparently wild-type revertant viruses without any residual intron sequence, and some viruses where the intron and NanoLuc gene were present; however, within the artificial intron we detected ≤3 nucleotide deletions or mismatches in the 3’ region of the intron at the ends of homopolymeric runs. This could either be the result of selection for mutant intron sequences or limitations of the sequencing itself. For all panels: *P < 0.05, **P < 0.001 and ns = not significant.
(TIF)

S5 Fig PR8-NA-intNL virus stability during mouse infections. (A) Diagram of mouse passaging experiments. (B) Luciferase levels in mouse lung homogenates from serial PR8-NA-intNL virus infections (1000PFU, 3d passages) in BL/6 mice (mean with SD, n = 3 mice per group); un, uninfected. (C) Titers in mouse lung homogenates from serial PR8-NA-intNL virus infections (1000PFU, 3d passages) in BL/6 mice (mean with SD, n = 3 mice per group). (D) Luciferase levels from infections (MOI = 0.001, multicycle, 24h) on MDCK cells using PR8-NA-intNL virus (passage 0) and mouse lung homogenates from serial PR8-NA-intNL virus infections (1000PFU, 3d passages) in BL/6 mice (mean with SD, n = 3 mice per group).
(TIF)

S6 Fig Determining PR8-NP-intNL mRNA splicing rates and virus stability in cell culture. (A) Top: Diagram of “NP-int/NP” (yellow) and “NP-int only” (green) dye-based qPCR primer locations on PR8 NP-intNL mRNAs. Bottom, left: Absolute standard curve detecting “NP-int/NP” and “NP-int only” sequences from a plasmid containing an PR8 NP intron-containing segment. Bottom, right: Absolute standard curve detecting “NP-int/NP” and “NP-int only” sequences from a plasmid containing an PR8 NP segment. (B) Top: Luciferase levels from infections (MOI = 0.001, multicycle, 24h) on MDCK cells using PR8-NP-intNL virus (passage 0) and supernatants from serial PR8-NP-intNL virus infections (MOI = 0.001, multicycle, 72h passages) on MDCK cells (mean with SD, n = 3 independent experiments, one-way ANOVA with Dunnett’s multiple comparisons test relative to passage 0). Bottom: RT-PCR of WT PR8 virus (yellow) and PR8-NP-intNL virus (green) segment 5/NP passages 0 to 10 from serial PR8-NP-intNL virus infections (MOI = 0.001, multicycle, 72h passages) on MDCK cells (representative of three independent experiments). ^#^After passage 10, viruses from infection supernatants were plaque purified and the intron-containing viral genomic segment was sequenced via Sanger sequencing. In all cases we detected apparently wild-type revertant viruses that did not harbor any residual intron sequence. For all panels: *P < 0.05, **P < 0.001 and ns = not significant.
(TIF)

S7 Fig Wyo/03-NA-intNL virus growth kinetics on MDCK cells. Growth kinetics of WT Wyo/03 and Wyo/03-NA-intNL virus infections (MOI = 0.0001, multicycle) on MDCK cells, measured using HA assays (mean with SD, n = 3 independent experiments). The downward error bar for the NA-intNL 72h data point could not be plotted on a log scale.
(TIF)

S8 Fig Constitutively spliced artificial intron sequence. (TIF)

S9 Fig NanoLuc-encoding artificial intron sequence. (TIF)

S10 Fig The full and unprocessed Western blot exposures corresponding to the composite images in Figs 1, 3 and 6. (A) Uncropped Western blots from Fig 1F. (B) Uncropped Western blots from Fig 3E. (C) Uncropped Western blots from Fig 6E. For all panels: red box, cropping in figure panel; white arrow, membrane cut.
(TIF)

S1 Table Inserting splicing environments into non-splicing influenza virus segments using silent mutations. (TIF)

S2 Table Primer and probe sequences for PR8 probe-based qPCR. (TIF)

S3 Table Primer sequences for PR8 dye-based qPCR. (TIF)
