# Supplementary figures and images for: Influenza A virus segments five and six can harbor artificial introns allowing expanded coding capacity
Source: PLoS Pathog. 2021 Sep 27;17(9):e1009951. doi: 10.1371/journal.ppat.1009951 (PMC8496794; doi:10.1371/journal.ppat.1009951)

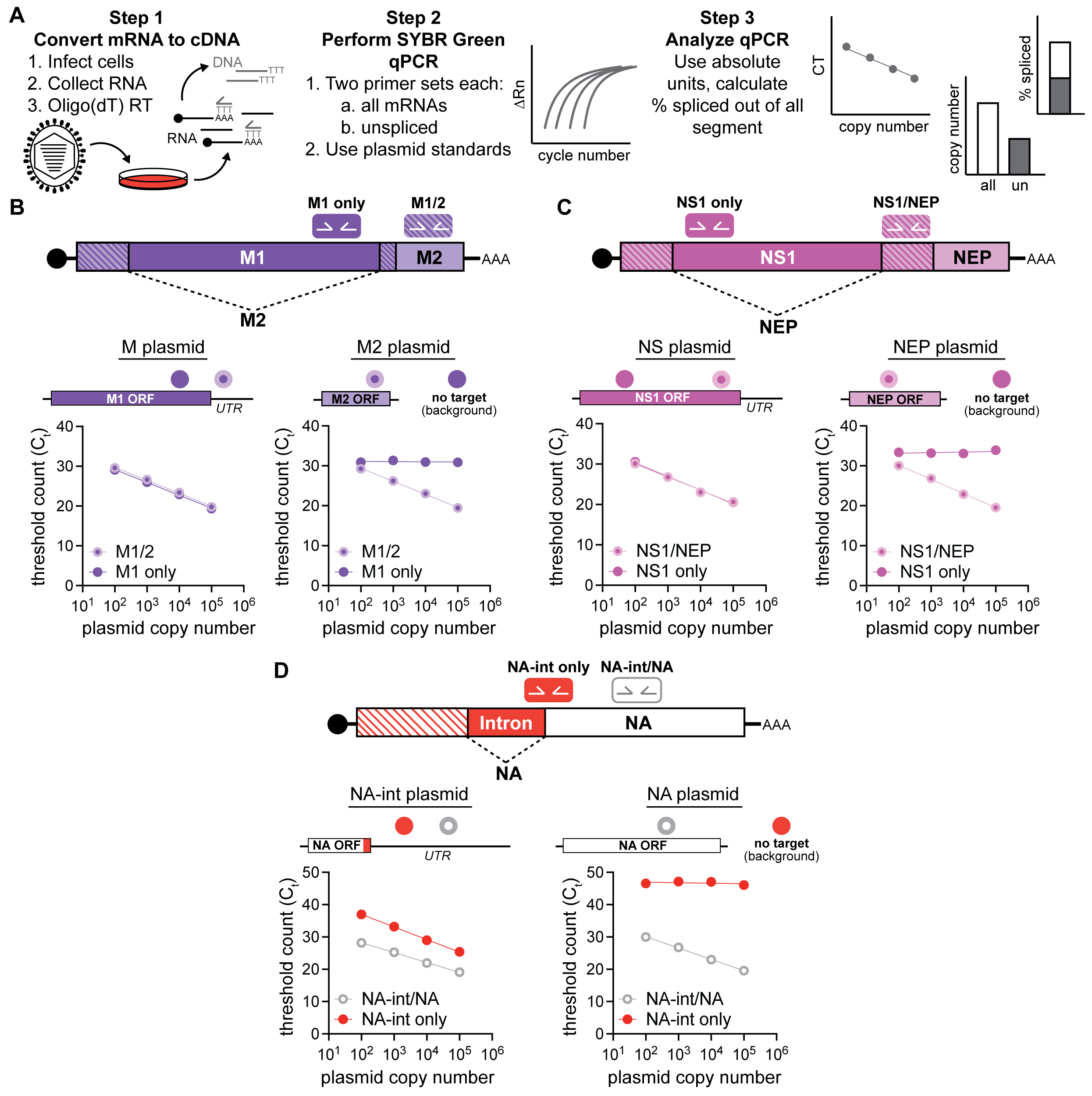

Supplement: S1 Fig — (A) Diagram of two-step RT-qPCR used to determine splicing rates. First, RNA was collected from infected cells/tissue and reverse-trancribed into DNA using oligo(dT) primers to select for mRNAs. Next, SYBR Green-based qPCR was performed using primers that targeted either 1) all mRNAs derived from an IAV segment, or 2) specifically unspliced mRNAs from that segment. Absolute values for the mRNA transcript copy numbers were determined using a standard curve of known plasmid concentrations encoding the segment of interest. Finally, using the generated standard curve, transcript copy numbers were determined for both 1) all mRNAs and 2) unspliced (un) mRNAs derived from the segment of interest and used to determine what percent of all transcripts from one segment were spliced. (B) Top: Diagram of “M1/2” (striped) and “M1 only” (solid) dye-based qPCR primer locations on PR8 M mRNAs. Bottom, left: Absolute standard curve detecting “M1/2” and “M1 only” sequences from a plasmid containing the PR8 M segment. Bottom, right: Absolute standard curve detecting “M1/2” and “M1 only” sequences from a plasmid containing the PR8 M2 ORF. UTR, untranslated region. (C) Top: Diagram of “NS1/NEP” (striped) and “NS1 only” (solid) dye-based qPCR primer locations on PR8 NS mRNAs. Bottom, right: Absolute standard curve detecting “NS1/NEP” and “NS1 only” sequences from a plasmid containing the PR8 NS segment. Bottom, left: Absolute standard curve detecting “NS1/NEP” and “NS1 only” sequences from a plasmid containing the PR8 NEP ORF. (D) Top: Diagram of “NA-int/NA” (white) and “NA-int only” (red) dye-based qPCR primer locations on PR8 NA-intron mRNAs. Bottom, left: Absolute standard curve detecting “NA-int/NA” and “NA-int only” sequences from a plasmid containing the PR8 NA intron-containing segment. Bottom, right: Absolute standard curve detecting “NA-int/NA” and “NA-int only” sequences from a plasmid containing the PR8 NA segment. (TIF) [file ppat.1009951.s001.tif]

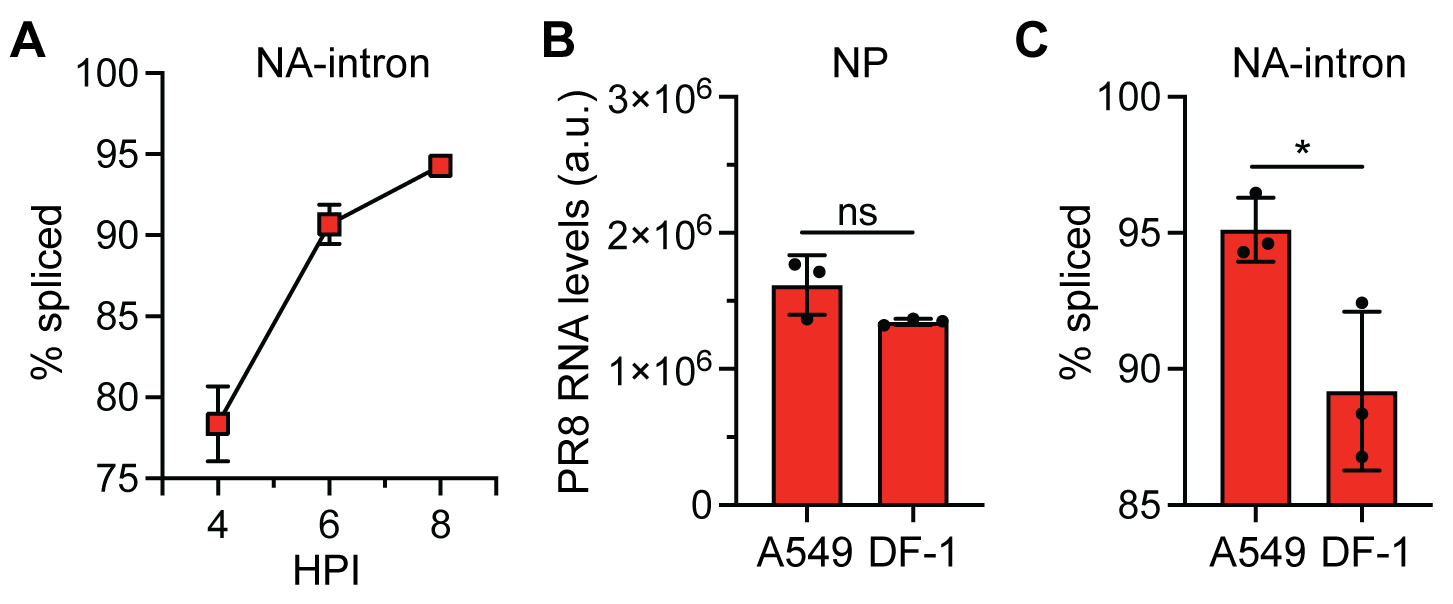

Supplement: S2 Fig — (A) PR8 segment 6/NA-intron mRNA splicing rates over time from PR8-NA-intron virus infections (MOI = 2, single cycle) on MDCK cells, measured using two-step RT-qPCR (mean with SD, n = 3 independent experiments). (B) PR8 NP RNA levels from PR8-NA-intron virus infections (MOI = 2, single cycle, 8h) on human lung A549 and avian embryonic DF-1 cells, measured using one-step RT-qPCR (mean with SD, n = 3 independent experiments, unpaired Student’s t-test). Arbitrary units (a.u.) determined relative to 18S. (C) PR8 segment 6/NA-intron mRNA splicing rates during PR8-NA-intron virus infections (MOI = 2, single cycle, 8h) on human lung A549 and avian embryonic DF-1 cells, measured using two-step RT-qPCR (mean with SD, n = 3 independent experiments, unpaired Student’s t-test). For all panels: *P < 0.05, **P < 0.001 and ns = not significant. (TIF) [file ppat.1009951.s002.tif]

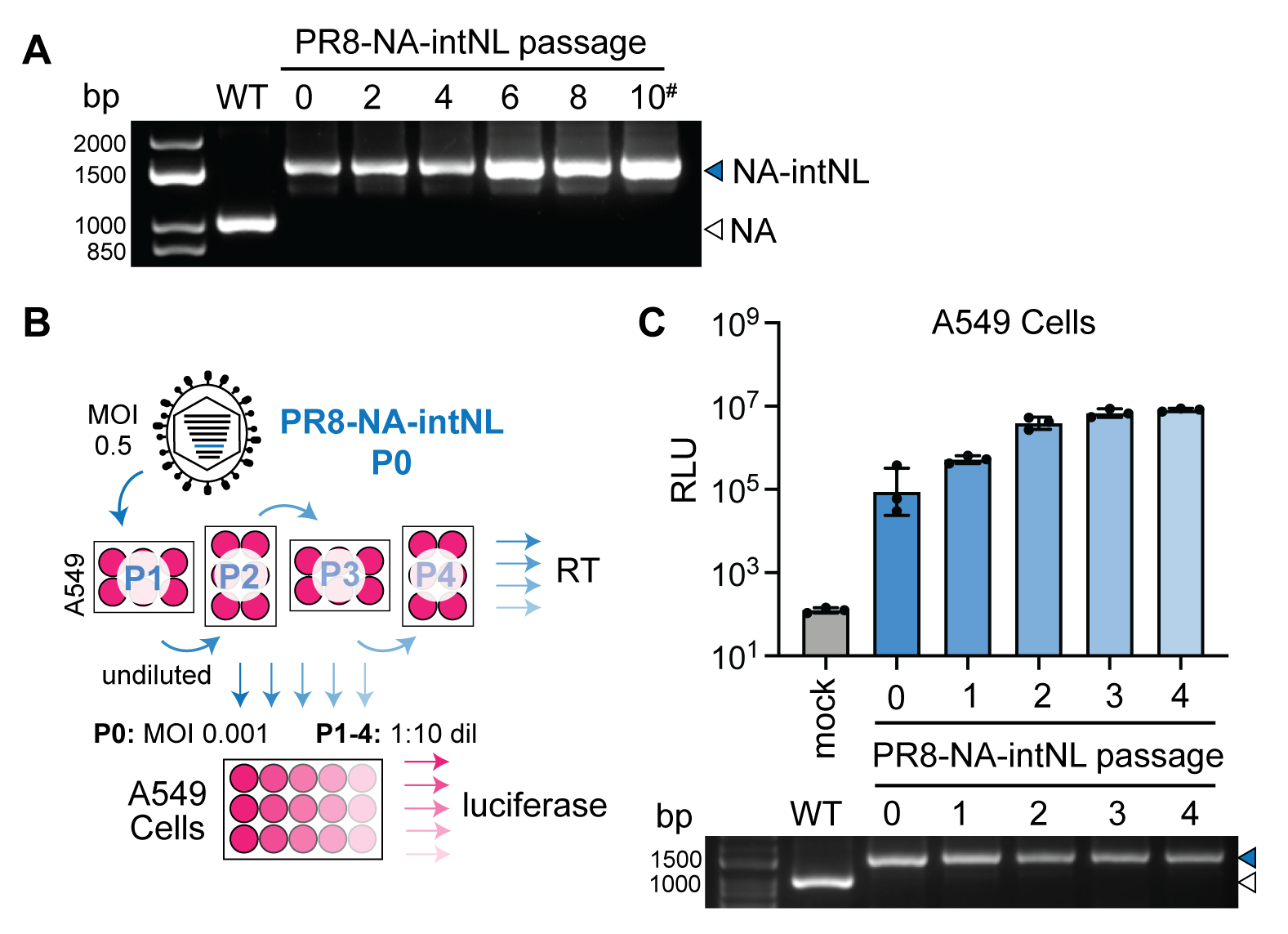

Supplement: S3 Fig — (A) RT-PCR of WT PR8 virus (white) and PR8-NA-intNL virus (blue) segment 6/NA passages 0 to 10 from serial PR8-NA-intNL virus infections (MOI = 0.001, multicycle, 72h passages) on MDCK cells (representative of three independent experiments). #After passage 10, viruses from infection supernatants were plaque purified and the intron-containing viral genomic segment was sequenced via Sanger sequencing. In all cases the intron and NanoLuc gene were present; however, within the artificial intron we detected ≤3 nucleotide deletions or mismatches in the 3’ region of the intron at the ends of homopolymeric runs. This could either be the result of selection for mutant intron sequences or limitations of the sequencing itself. (B) Diagram of A549 cell passaging experiments. (C) Top: Luciferase levels from infections (multicycle, 24h) on A549 cells using PR8-NA-intNL virus (passage 0) and supernatants from serial PR8-NA-intNL virus infections (MOI = 0.5, multicycle, 72h passages) on A549 cells (mean with SD, n = 3 independent experiments). Bottom: RT-PCR of WT PR8 virus (white) and PR8-NA-intNL virus (blue) virus from passages 0 to 4 from serial PR8-NA-intNL virus infections (MOI = 0.5, multicycle, 72h passages) on A549 cells (representative of three independent experiments). (TIF) [file ppat.1009951.s003.tif]

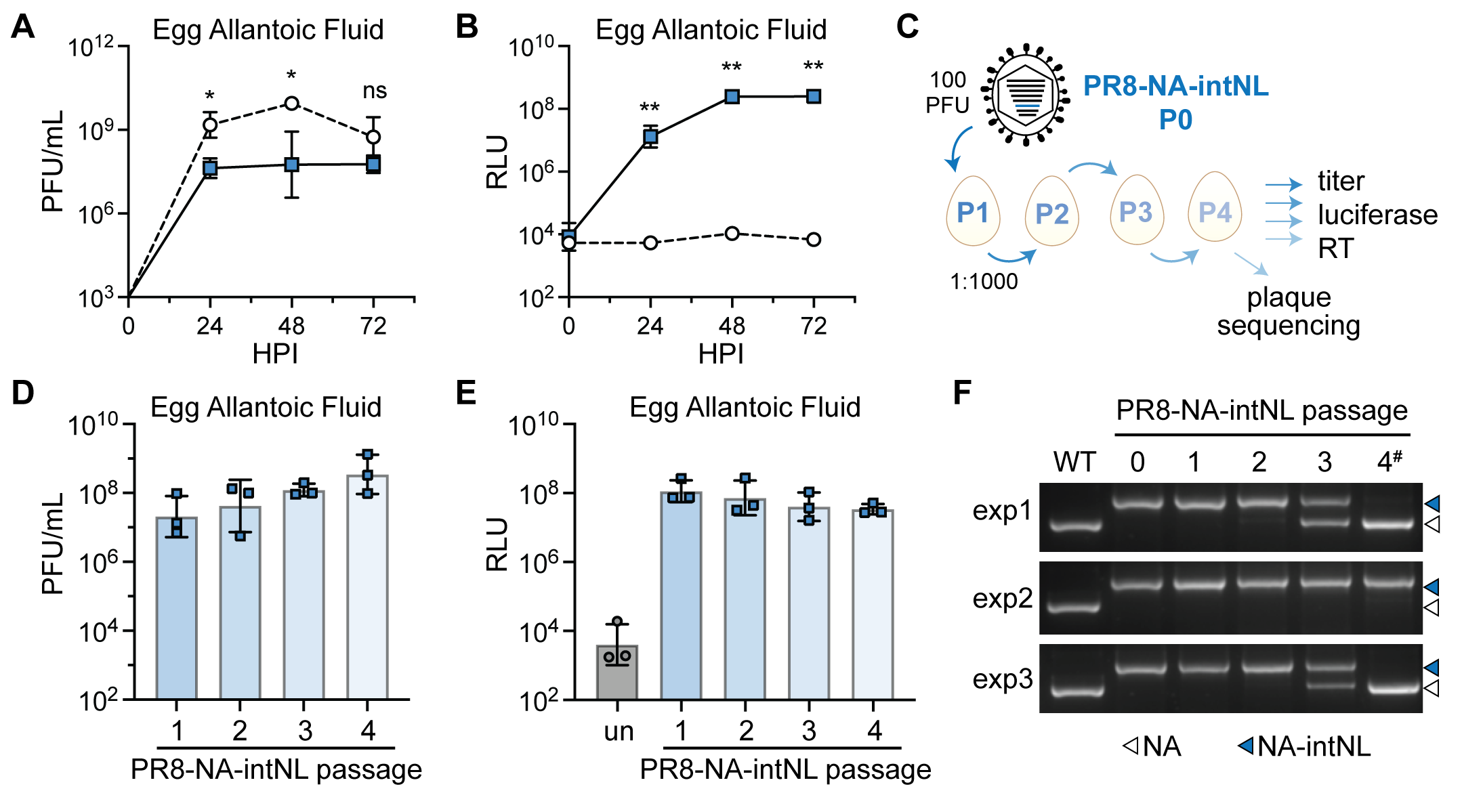

Supplement: S4 Fig — (A) Growth kinetics of WT PR8 and PR8-NA-intNL virus infections (100PFU) in embryonated chicken eggs, measured using plaque assays (mean with SD, n = 3 eggs per group, unpaired Student’s t-test relative to WT). (B) Luciferase levels in egg allantoic fluid from WT PR8 and PR8-NA-intNL virus infections (100PFU) in embryonated chicken eggs (mean with SD, n = 3 eggs per group, unpaired Student’s t-test relative to WT). (C) Diagram of egg passaging experiments. (D) Titers in egg allantoic fluid from serial PR8-NA-intNL virus infections (100PFU, 72h passages) in embryonated chicken eggs, measured using plaque assays (mean with SD, n = 3 eggs per group). (E) Luciferase levels in egg allantoic fluid from serial PR8-NA-intNL virus infections (100PFU, 72h passages) in embryonated chicken eggs (mean with SD, n = 3 eggs per group); un, uninfected. (F) RT-PCR of WT PR8 virus (white) and PR8-NA-intNL virus (blue) segment 6/NA from egg passages 0 to 4; exp, independent experiment. #After passage 4, viruses from infected egg allantoic fluid were plaque purified and the intron-containing viral genomic segment was sequenced via Sanger sequencing. In all cases we detected a mixed population within one stock, with some apparently wild-type revertant viruses without any residual intron sequence, and some viruses where the intron and NanoLuc gene were present; however, within the artificial intron we detected ≤3 nucleotide deletions or mismatches in the 3’ region of the intron at the ends of homopolymeric runs. This could either be the result of selection for mutant intron sequences or limitations of the sequencing itself. For all panels: *P < 0.05, **P < 0.001 and ns = not significant. (TIF) [file ppat.1009951.s004.tif]

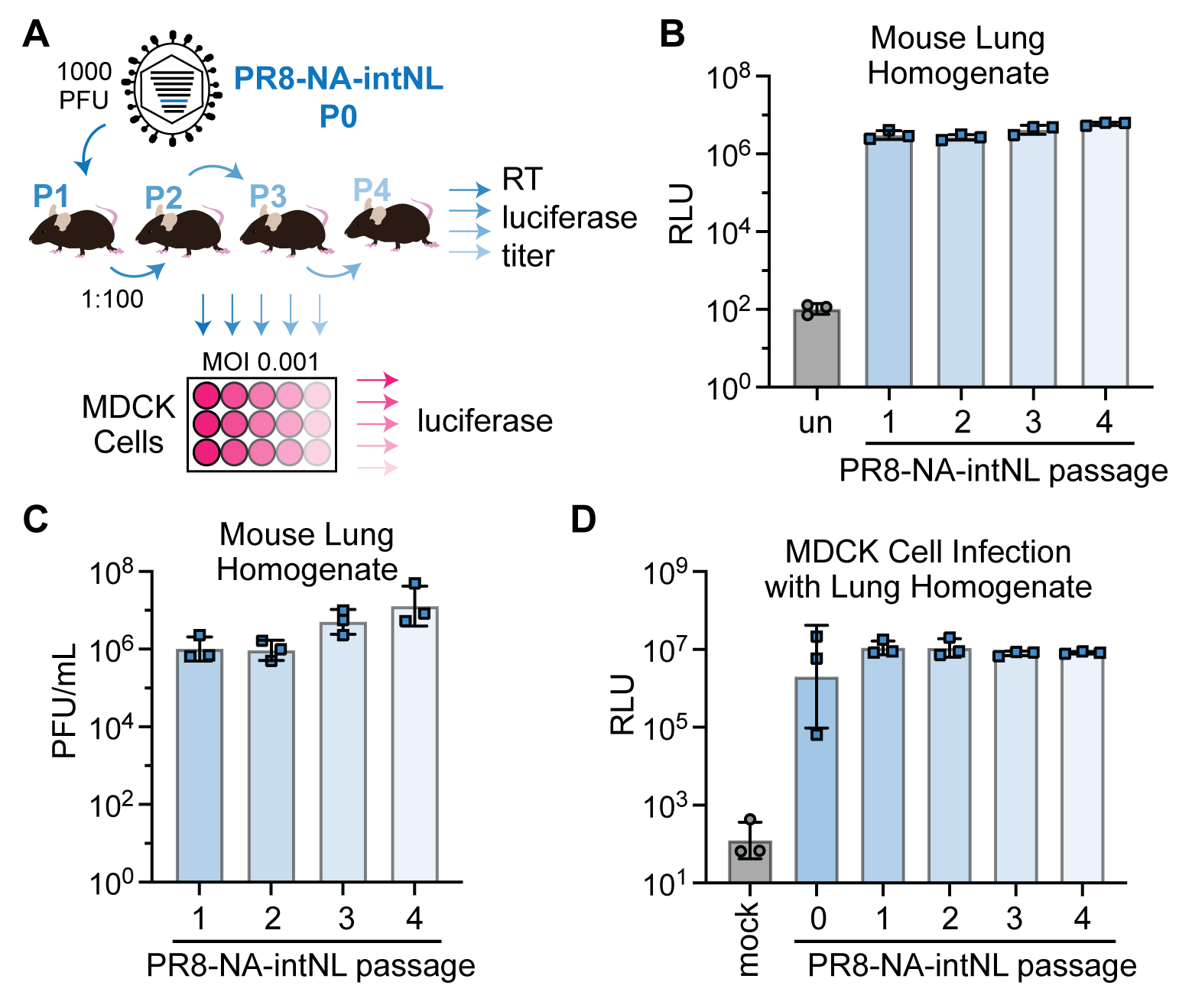

Supplement: S5 Fig — (A) Diagram of mouse passaging experiments. (B) Luciferase levels in mouse lung homogenates from serial PR8-NA-intNL virus infections (1000PFU, 3d passages) in BL/6 mice (mean with SD, n = 3 mice per group); un, uninfected. (C) Titers in mouse lung homogenates from serial PR8-NA-intNL virus infections (1000PFU, 3d passages) in BL/6 mice (mean with SD, n = 3 mice per group). (D) Luciferase levels from infections (MOI = 0.001, multicycle, 24h) on MDCK cells using PR8-NA-intNL virus (passage 0) and mouse lung homogenates from serial PR8-NA-intNL virus infections (1000PFU, 3d passages) in BL/6 mice (mean with SD, n = 3 mice per group). (TIF) [file ppat.1009951.s005.tif]

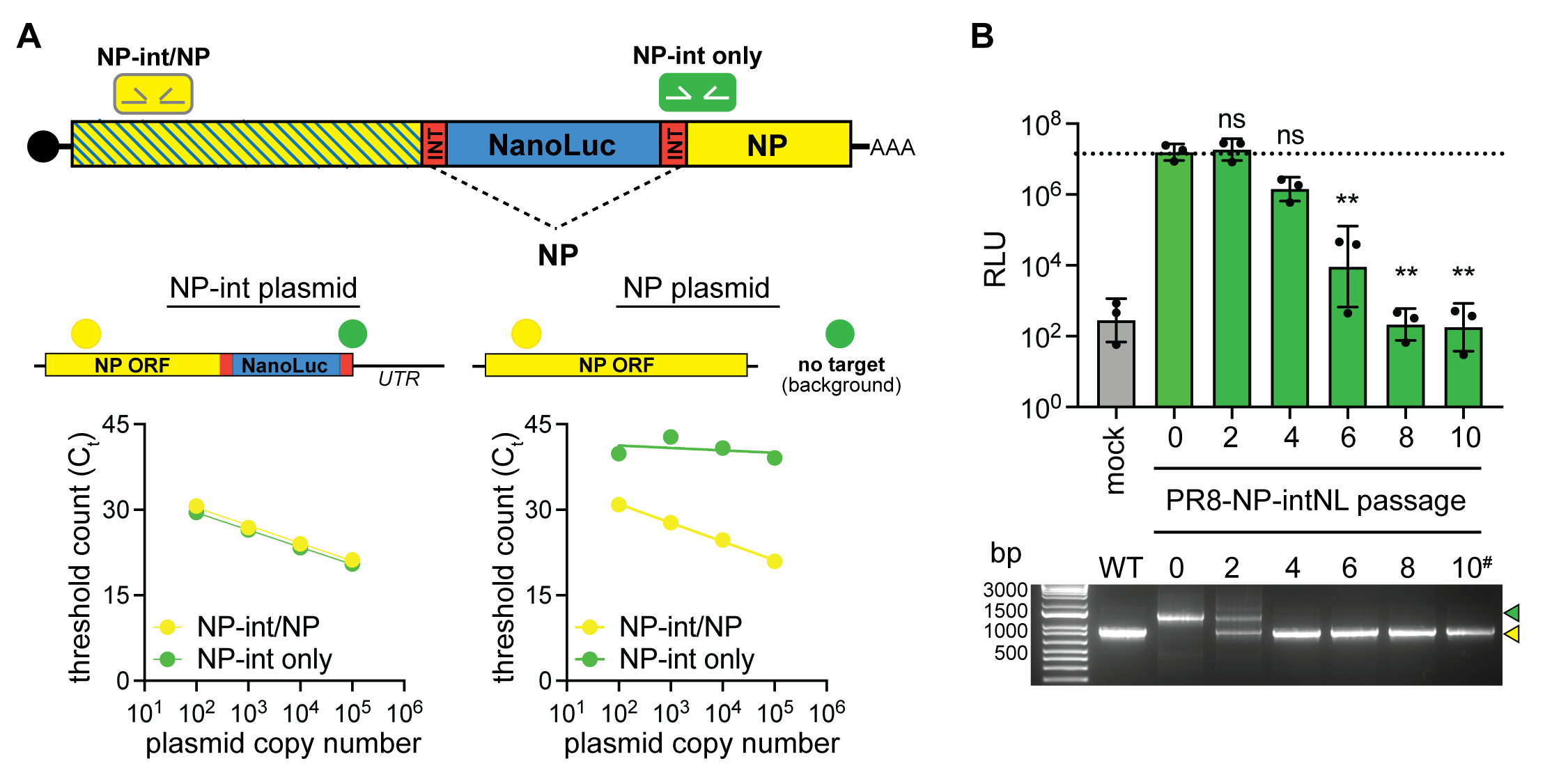

Supplement: S6 Fig — (A) Top: Diagram of “NP-int/NP” (yellow) and “NP-int only” (green) dye-based qPCR primer locations on PR8 NP-intNL mRNAs. Bottom, left: Absolute standard curve detecting “NP-int/NP” and “NP-int only” sequences from a plasmid containing an PR8 NP intron-containing segment. Bottom, right: Absolute standard curve detecting “NP-int/NP” and “NP-int only” sequences from a plasmid containing an PR8 NP segment. (B) Top: Luciferase levels from infections (MOI = 0.001, multicycle, 24h) on MDCK cells using PR8-NP-intNL virus (passage 0) and supernatants from serial PR8-NP-intNL virus infections (MOI = 0.001, multicycle, 72h passages) on MDCK cells (mean with SD, n = 3 independent experiments, one-way ANOVA with Dunnett’s multiple comparisons test relative to passage 0). Bottom: RT-PCR of WT PR8 virus (yellow) and PR8-NP-intNL virus (green) segment 5/NP passages 0 to 10 from serial PR8-NP-intNL virus infections (MOI = 0.001, multicycle, 72h passages) on MDCK cells (representative of three independent experiments). #After passage 10, viruses from infection supernatants were plaque purified and the intron-containing viral genomic segment was sequenced via Sanger sequencing. In all cases we detected apparently wild-type revertant viruses that did not harbor any residual intron sequence. For all panels: *P < 0.05, **P < 0.001 and ns = not significant. (TIF) [file ppat.1009951.s006.tif]

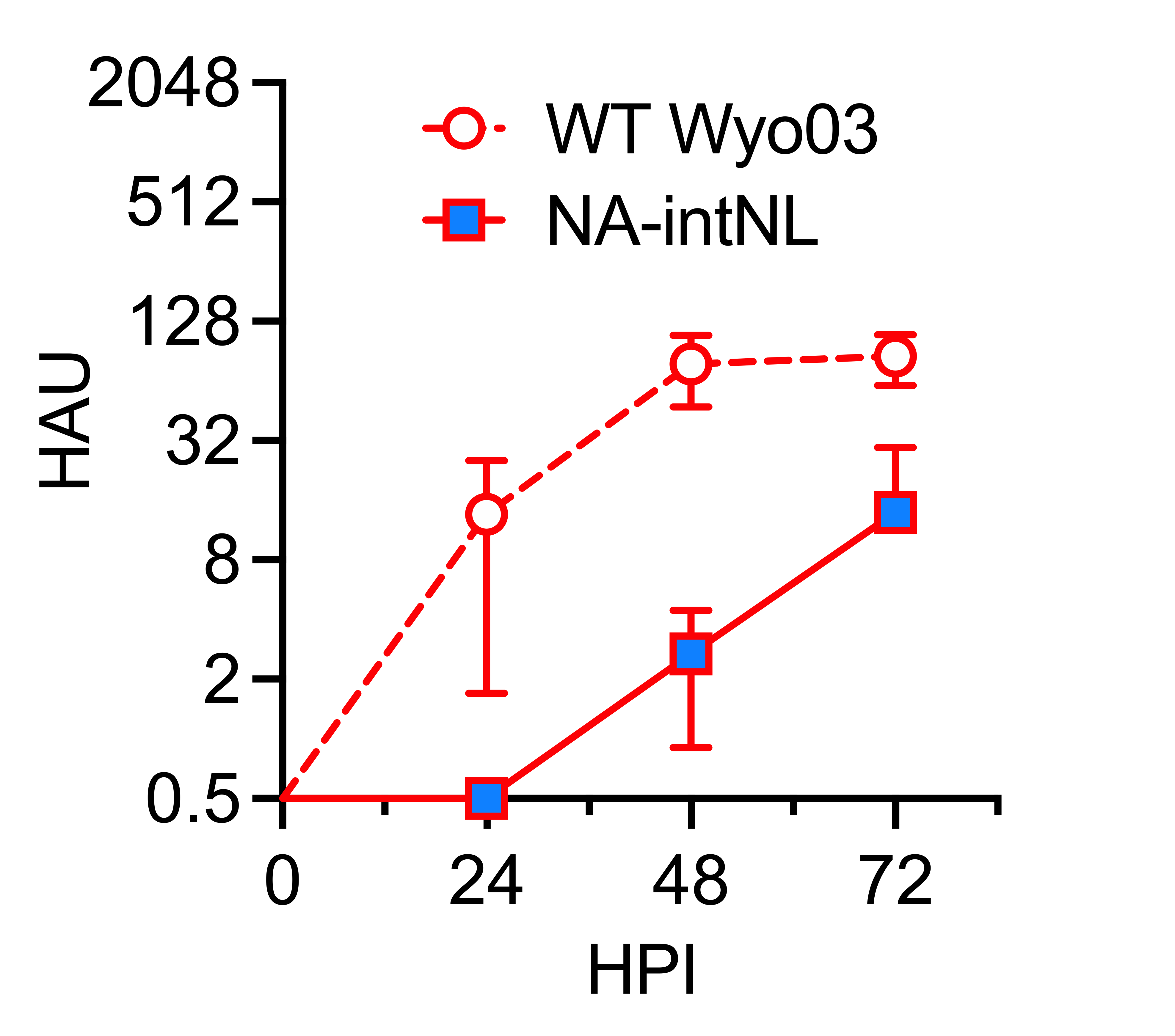

Supplement: S7 Fig — Growth kinetics of WT Wyo/03 and Wyo/03-NA-intNL virus infections (MOI = 0.0001, multicycle) on MDCK cells, measured using HA assays (mean with SD, n = 3 independent experiments). The downward error bar for the NA-intNL 72h data point could not be plotted on a log scale. (TIF) [file ppat.1009951.s007.tif]

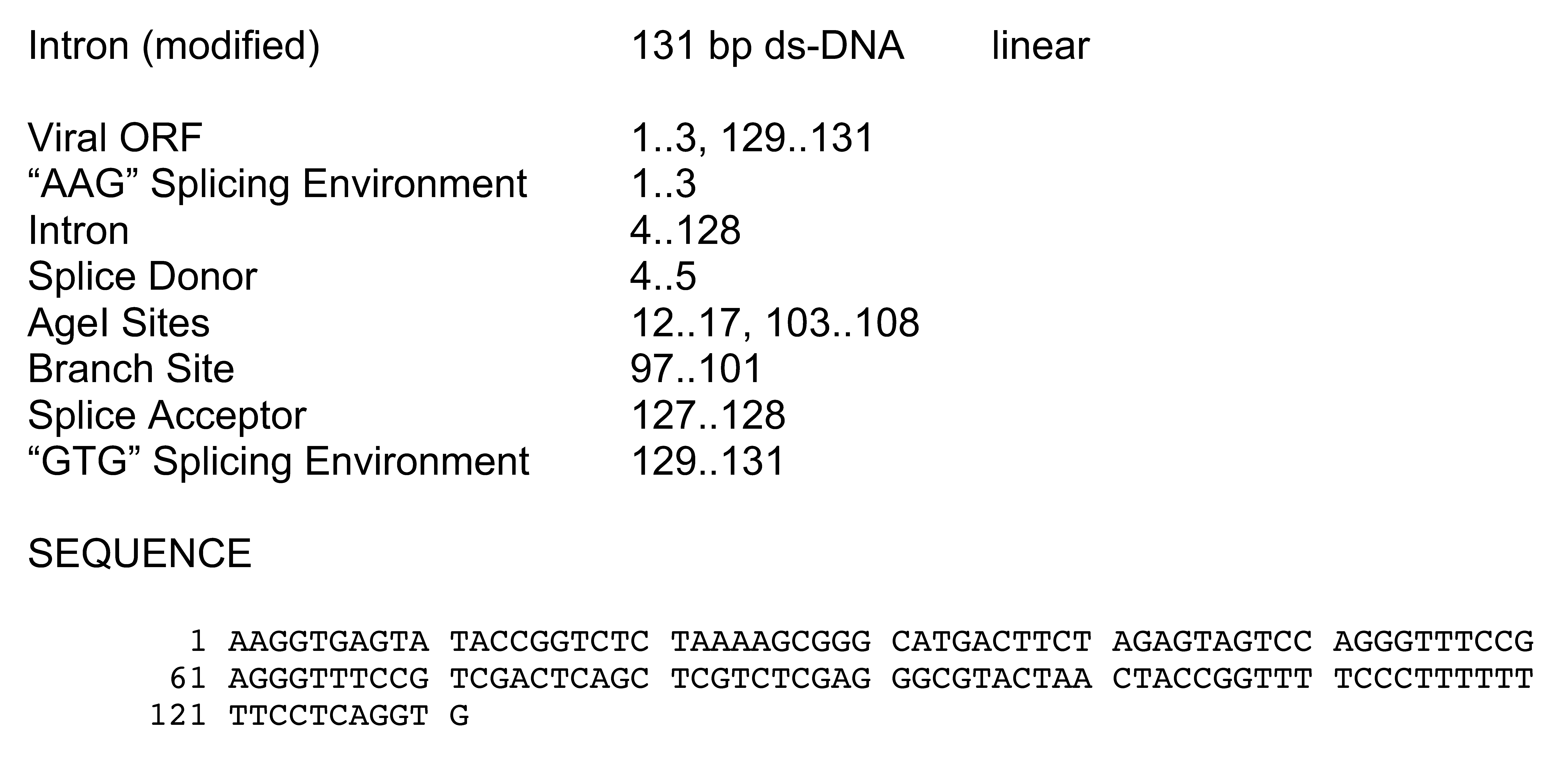

Supplement: S8 Fig — (TIF) [file ppat.1009951.s008.tif]

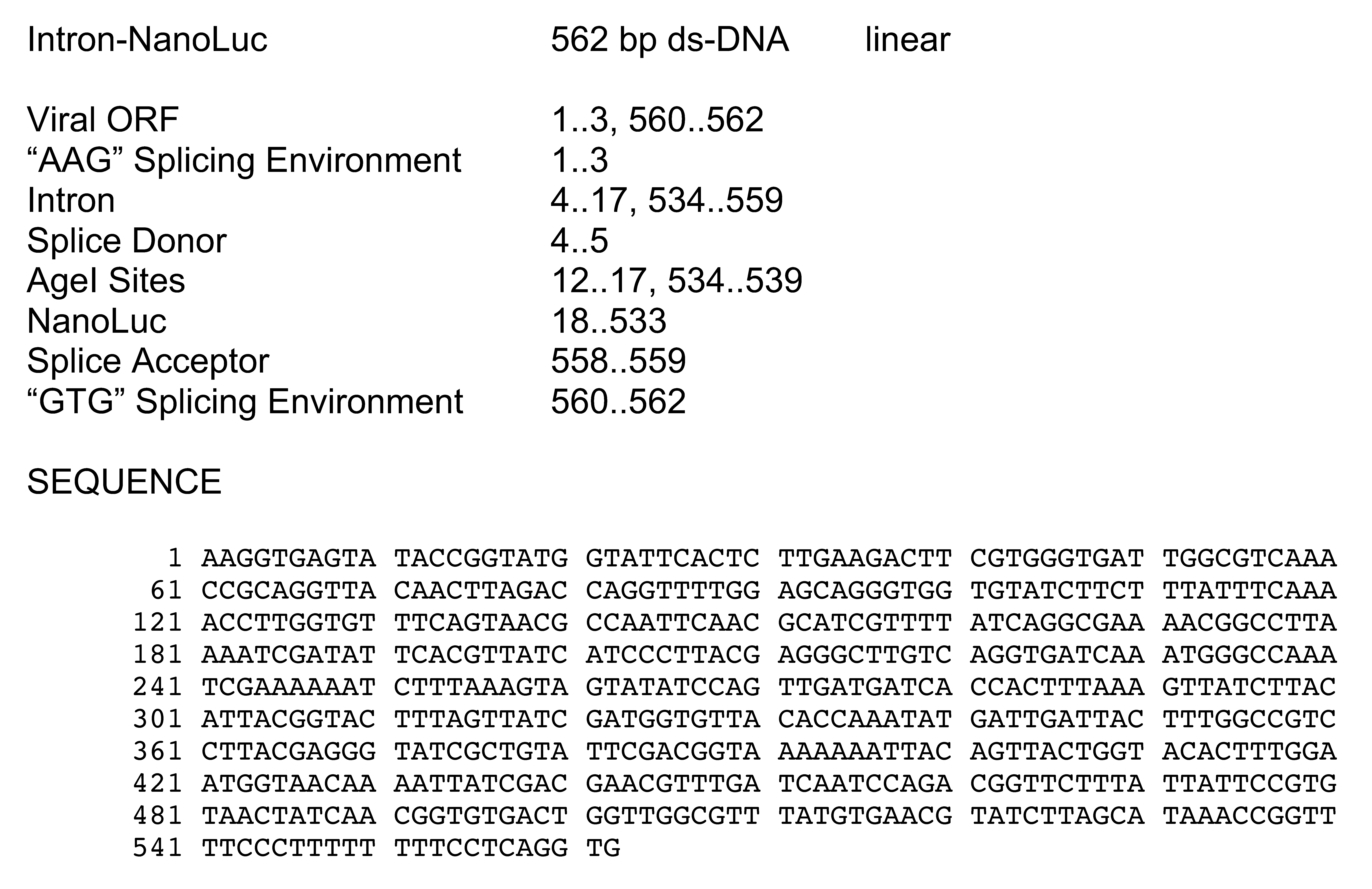

Supplement: S9 Fig — (TIF) [file ppat.1009951.s009.tif]

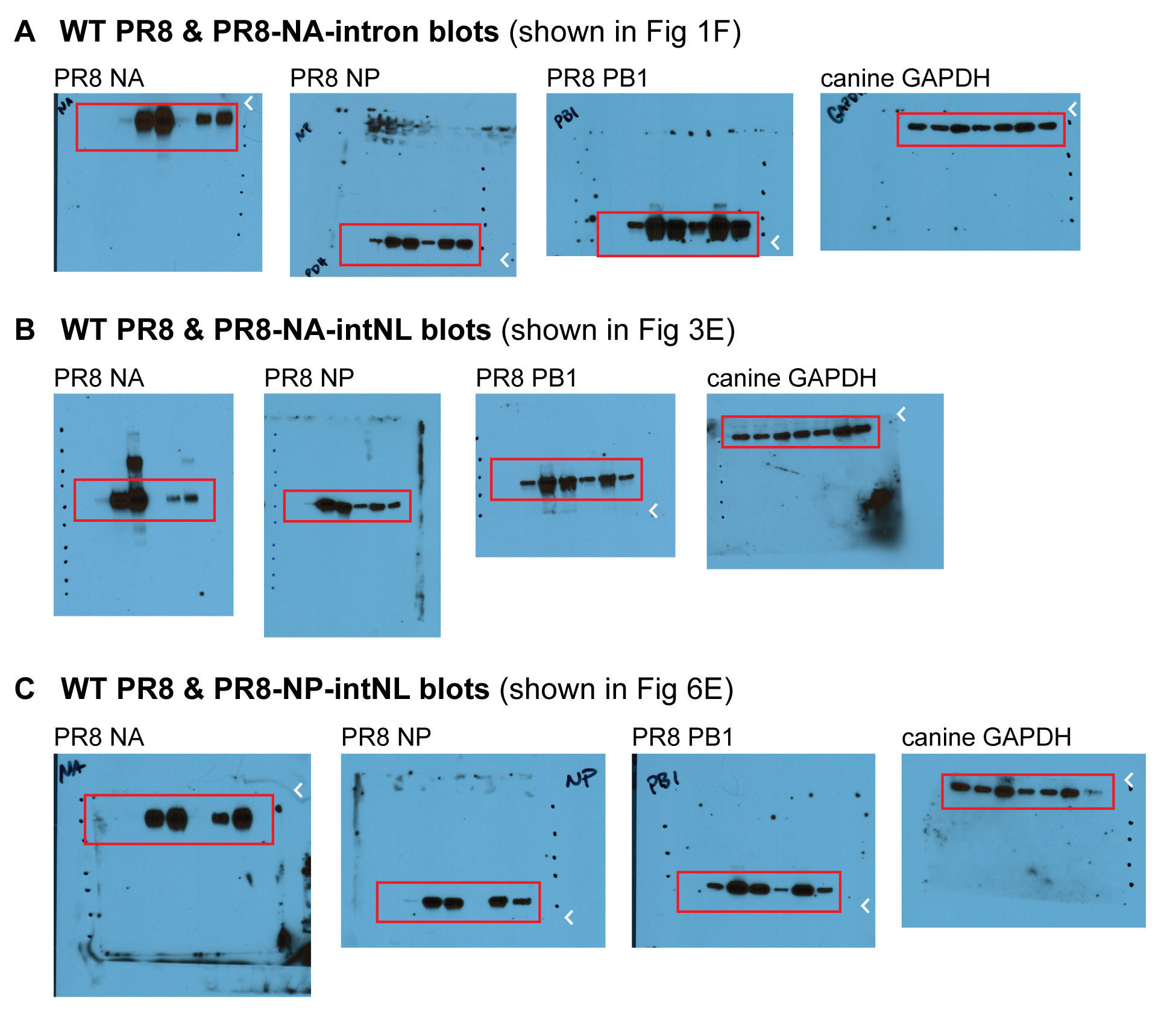

Supplement: S10 Fig — (A) Uncropped Western blots from Fig 1F. (B) Uncropped Western blots from Fig 3E. (C) Uncropped Western blots from Fig 6E. For all panels: red box, cropping in figure panel; white arrow, membrane cut. (TIF) [file ppat.1009951.s010.tif]

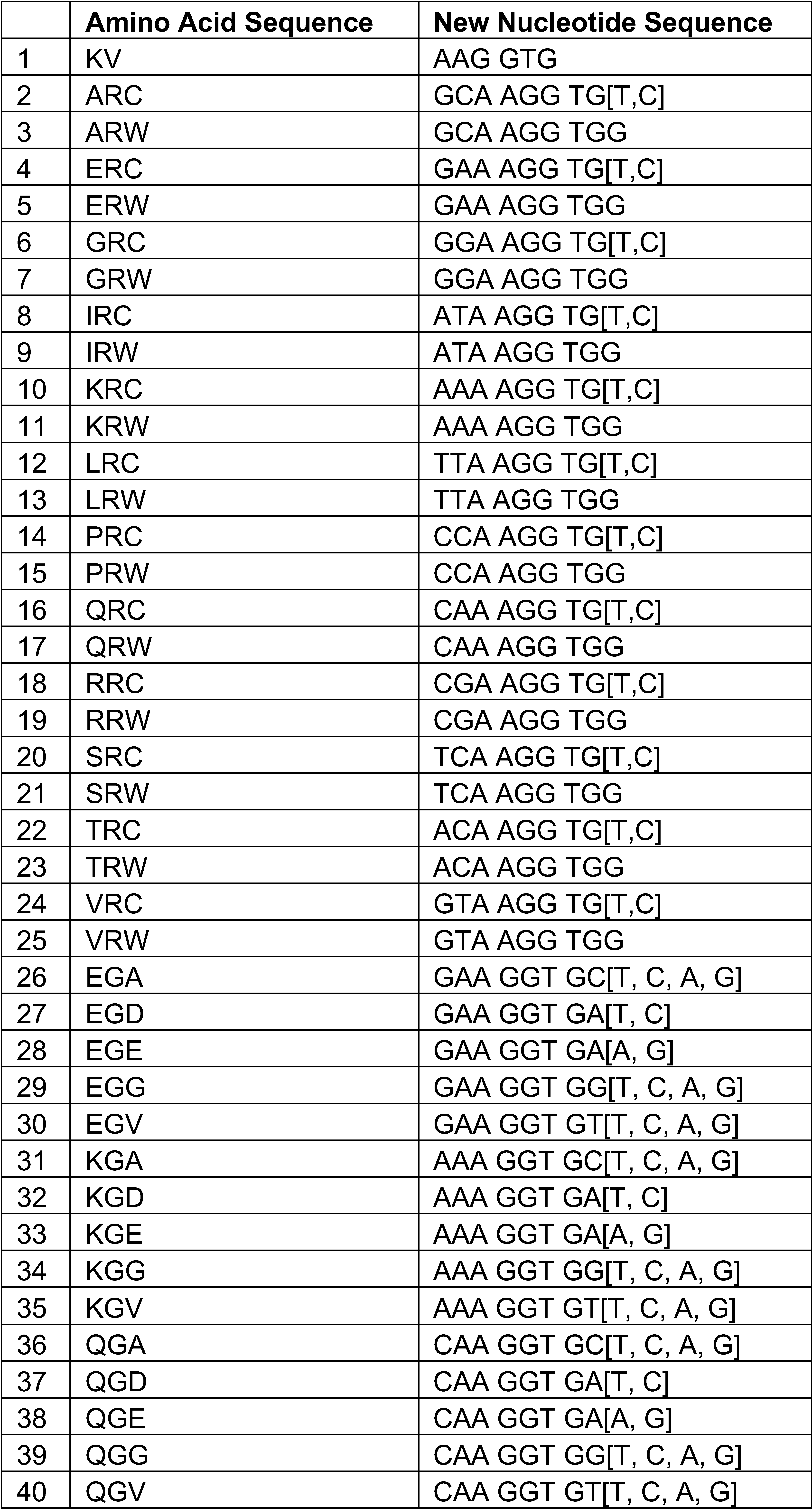

Supplement: S1 Table — (TIF) [file ppat.1009951.s011.tif]

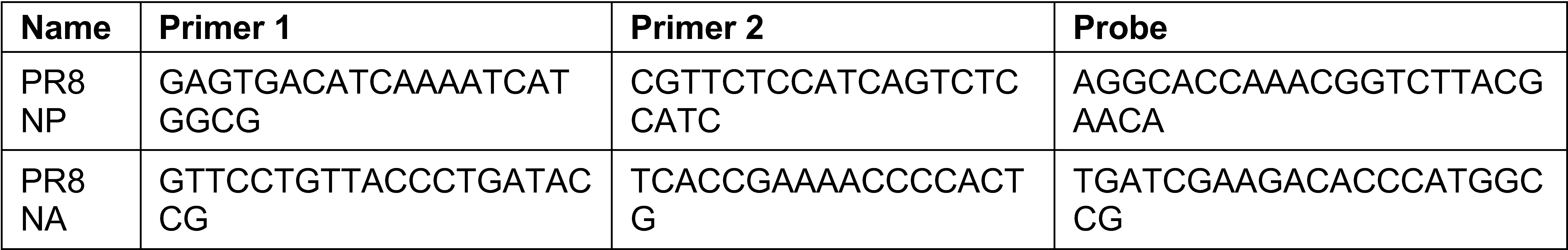

Supplement: S2 Table — (TIF) [file ppat.1009951.s012.tif]

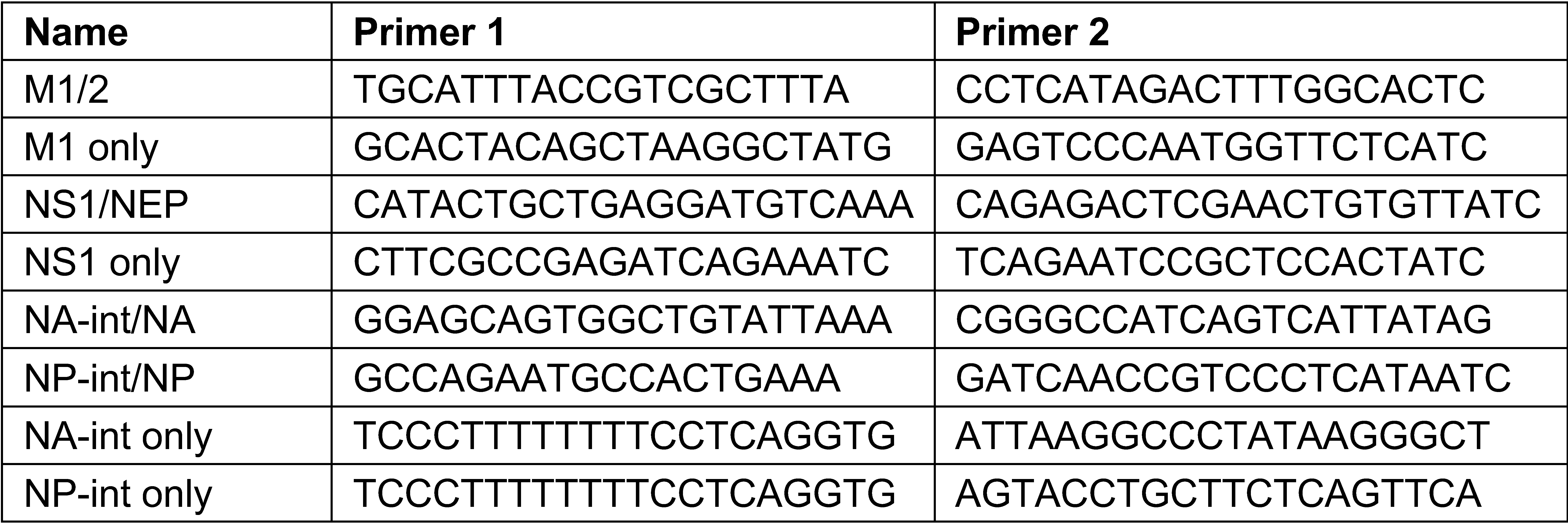

Supplement: S3 Table — (TIF) [file ppat.1009951.s013.tif]
